# Transcriptomic profiling of immune cells in murine polymicrobial sepsis

**DOI:** 10.3389/fimmu.2024.1347453

**Published:** 2024-01-26

**Authors:** Atsushi Murao, Alok Jha, Monowar Aziz, Ping Wang

**Affiliations:** ^1^ Center for Immunology and Inflammation, The Feinstein Institutes for Medical Research, Manhasset, NY, United States; ^2^ Departments of Surgery and Molecular Medicine, Zucker School of Medicine at Hofstra/Northwell, Manhasset, NY, United States

**Keywords:** sepsis, single-cell RNA sequencing, neutrophil, macrophage, lymphocyte

## Abstract

**Introduction:**

Various immune cell types play critical roles in sepsis with numerous distinct subsets exhibiting unique phenotypes even within the same cell population. Single-cell RNA sequencing (scRNA-seq) enables comprehensive transcriptome profiling and unbiased cell classification. In this study, we have unveiled the transcriptomic landscape of immune cells in sepsis through scRNA-seq analysis.

**Methods:**

We induced sepsis in mice by cecal ligation and puncture. 20 h after the surgery, the spleen and peritoneal lavage were collected. Single-cell suspensions were processed using a 10× Genomics pipeline and sequenced on an Illumina platform. Count matrices were generated using the Cell Ranger pipeline, which maps reads to the mouse reference transcriptome, GRCm38/mm10. Subsequent scRNA-seq analysis was performed using the R package Seurat.

**Results:**

After quality control, we subjected the entire data set to unsupervised classification. Four major clusters were identified as neutrophils, macrophages, B cells, and T cells according to their putative markers. Based on the differentially expressed genes, we identified activated pathways in sepsis for each cell type. In neutrophils, pathways related to inflammatory signaling, such as NF-κB and responses to pathogen-associated molecular patterns (PAMPs), cytokines, and hypoxia were activated. In macrophages, activated pathways were the ones related to cell aging, inflammatory signaling, and responses to PAMPs. In B cells, pathways related to endoplasmic reticulum stress were activated. In T cells, activated pathways were the ones related to inflammatory signaling, responses to PAMPs, and acute lung injury. Next, we further classified each cell type into subsets. Neutrophils consisted of four clusters. Some subsets were activated in inflammatory signaling or cell metabolism, whereas others possessed immunoregulatory or aging properties. Macrophages consisted of four clusters, namely, the ones with enhanced aging, lymphocyte activation, extracellular matrix organization, or cytokine activity. B cells consisted of four clusters, including the ones possessing the phenotype of cell maturation or aging. T cells consisted of six clusters, whose phenotypes include molecular translocation or cell activation.

**Conclusions:**

Transcriptomic analysis by scRNA-seq has unveiled a comprehensive spectrum of immune cell responses and distinct subsets in the context of sepsis. These findings are poised to enhance our understanding of sepsis pathophysiology, offering avenues for targeting novel molecules, cells, and pathways to combat infectious diseases.

## Introduction

Sepsis is a critical infectious disease condition accompanied by organ dysfunction due to dysregulated host immune response to invading pathogens (1). It is estimated that 49 million people suffer from sepsis, resulting in 11 million deaths worldwide every year (2). Pathogen-associated molecular patterns (PAMPs), such as LPS, and damage-associated molecular patterns (DAMPs), including, but not limited to, extracellular cold-inducible RNA-binding protein (eCIRP), high mobility group box 1 (HMGB1), and histone H3, initiate immune responses through the stimulation of pattern recognition receptors (PRRs), leading to the activation of transcription factors, such as NF-κB, to induce inflammation (3–6). Different kinds of immune cells, such as neutrophils, macrophages, B cells, and T cells, coordinately play critical roles during sepsis (7). It is now known that each cell type consists of quite a few subsets (8). Those subsets exhibit a distinct phenotype reflected by genomic differences even within the same cell population (8). For instance, neutrophils, which were traditionally thought as a terminally differentiated single population, are now known to consist of many subsets, such as aged, antigen-presenting, intercellular adhesion molecule-1 (ICAM-1)-expressing, and low-density neutrophils and antigen-presenting aged neutrophils (APANs) in sepsis (9–13). T-cell and macrophage polarization is no longer highlighted as a simple Th1/Th2 and M1/M2 dichotomy as once thought (8). In addition, numerous cellular molecules change their status cell by cell in sepsis, contributing to the disease development (14). It should also be noted that the distribution and phenotype of cells differ between the organs in sepsis. Considering that immune cells mediate crosstalk between the site of infection and systemic inflammation in sepsis (15, 16), assessments of immune cell activities at the focus of infection along with that of remote organs are important to precisely delineate the pathophysiology of this deadly disorder.

Single-cell RNA sequencing (scRNA-seq) enables us to comprehensively screen the genetic status in individual cells and classify the cells into clusters in an unbiased way (17, 18). We are able to not only identify major cell populations, such as neutrophils, macrophages, and lymphocytes, but also further clusterize them into minor subsets unbiasedly (17). Moreover, based on the differentially expressed genes of the cluster, activated pathways can be statistically determined using specific algorithms, such as gene set enrichment analysis (GSEA) (19). A growing number of studies implement scRNA-seq to accelerate the understanding of different disorders (20–22). Furthermore, it also facilitates drug development and transition from the bench to the bedside (20, 21). In this study, we have delved into the transcriptomic landscape of immune cells in sepsis through scRNA-seq analysis. Using intraabdominal septic mice, we have revealed the frequency and status of neutrophils, macrophages, B cells, and T cells in the peritoneal cavity, the focus of infection, and spleen, a remote immune organ, simultaneously. We further classified those major cell populations into subtypes, which showed distinct characteristics in inflammatory response, cellular aging, and metabolism. These discoveries identifying new pathways offer future research areas in sepsis to advance our understanding of its pathophysiology, potentially leading to therapeutics targeting the novel molecules, cells, and pathways to address this deadly disease syndrome.

## Materials and methods

### Animals

Male C57BL/6 mice, 8–12 weeks old, were purchased from Charles River Laboratories (Wilmington, MA). The mice were housed in a temperature-controlled room with 12-h intermittent light and dark cycles and fed a standard mouse chow diet with drinking water. All animal experiments were performed following the National Institutes of Health Guide for the Care and Use of Laboratory Animals and were approved by our Institutional Animal Care and Use Committee (IACUC).

### Mouse model of sepsis

Polymicrobial sepsis was induced in mice by cecal ligation and puncture (CLP) (9). In brief, mice were anesthetized with 2% isoflurane inhalation, and a midline abdominal incision was created. The cecum was securely tied off with a 4–0 silk suture at 1 cm proximal from its distal extremity and then punctured twice using a 22-gauge needle to create two small through-and-through pores. Sham animals were subjected to a laparotomy without CLP. Following the surgery, 1 mL of normal saline was subcutaneously (*s.c.*) injected to avoid surgery-induced dehydration and 0.05 mg/kg buprenorphine was *s.c*. injected as an analgesic. 20 h after the surgery, the peritoneal lavage and spleen were harvested. Samples were pooled from three mice per group.

### Isolation of peritoneal cells and splenocytes

Peritoneal lavage was collected by washing with cold PBS and centrifuged at 300 × g for 5 min at 4°C. The cell pellets were then resuspended into complete RPMI medium. Spleens were grounded and passed through a 70-μm nylon cell strainer. The splenocyte suspension was centrifuged at 300 × g for 5 min at 4°C. The cell pellet was suspended in 1-mL red blood cell (RBC) lysing buffer (BD Biosciences) to lyse the RBCs in the suspension, followed by washing of the cells with PBS. The cell pellets were then resuspended into complete RPMI medium.

### Droplet-based scRNA-seq and genomic mapping

Cells from the peritoneal cavity and spleen of sham and CLP mice were sorted for scRNA-seq using the 10× Genomics Chromium platform. Library preparation was conducted according to the recommended protocol for the Next GEM Single Cell 3' Library Kit v3.1 (no. 1000121; 10× Genomics). Libraries were sequenced on the Illumina NextSeq 2000 sequencing platform to a mean depth of approximately 40,000 reads per cell. The Cell Ranger count pipeline (v6.0.0, 10× Genomics) was used to align FASTQs to the mouse reference genome (gex-mm10-2020-A, 10× Genomics) and produce digital gene-cell count matrices and to perform quality control of the mapping results.

Primary assessment with Cell Ranger for cells from the peritoneal cavity of septic mice reported 6,596 cells with a median of 4,972 unique molecular identifiers per cell and a median of 1,122 genes per cell at 75.3% sequence saturation, with a mean of 46,465 reads per cell. Primary assessment with Cell Ranger for cells from the spleen of septic mice reported 7,423 cells with a median of 3,483 unique molecular identifiers per cell and a median of 1,310 genes per cell at 72.0% sequence saturation, with a mean of 41,567 reads per cell. Primary assessment with Cell Ranger for cells from the peritoneal cavity of sham mice reported 5,462 cells with a median of 12,014 unique molecular identifiers per cell and a median of 2,984 genes per cell at 56.2% sequence saturation, with a mean of 46,645 reads per cell. Primary assessment with Cell Ranger for cells from the spleen of sham mice reported 8,148 cells with a median of 3,741 unique molecular identifiers per cell and a median of 1,362 genes per cell at 64.4% sequence saturation, with a mean of 35,614 reads per cell.

### Single-cell sequencing quantification

DoubletFinder was used to identify doublets. After filtering out doublets, Seurat v4.1.3 was used to filter out cells expressing less than 200 genes and all cells with more than 5% mitochondrial genes. Among cells from the peritoneal cavity of septic mice, 6,164 singlets were kept and 432 cells were removed through quality control. Among cells from the spleen of septic mice, 3,137 singlets were kept and 4,286 cells were removed through quality control. Among cells from the peritoneal cavity of sham mice, 4,288 singlets were kept and 1,174 cells were removed through quality control. Among cells from the spleen of sham mice, 5,367 singlets were kept and 2,781 cells were removed through quality control. Gene expression normalization and cell clustering were done using the SCTransform pipeline (23) with percent mitochondrial reads regressed out and batch effects corrected using Harmony (24). To apply the SCTransform process implemented in Seurat (25), we began by using the “SCTransform” function to normalize the data and regress out mitochondrial mapping percentage as a confounding source of variation, as is standard (23). We then used the “RunPCA” function to run a principal component analysis (PCA), using the variable features (genes) to compute the principal components (PCs). Next, we used Harmony to correct for batch effects (based on sequencing run and the origin of the cells from peritoneal cavity vs. spleen), with the SCTransformed data used as the input, for a maximum of 50 rounds of Harmony (each involving at most 100 rounds of clustering and a correction step). Subsequently, we used the “RunUMAP” function to utilize the Uniform Manifold Approximation and Projection (UMAP) approach to dimensionality reduction, using the first 20 dimensions of the harmonized data as the input features to the UMAP. Finally, the “FindNeighbors” function was used to compute the 20 nearest neighbors for a given dataset based on the first 20 dimensions of the harmonized data, and the “FindClusters” function was applied to identify clusters of cells by a shared nearest neighbor (SNN) modularity optimization-based clustering algorithm.

### Gene set enrichment analysis

A gene set enrichment analysis (GSEA) was performed using fgsea 1.26.0, an R package. In this case, Molecular Signatures Database (MSigDB) v2022.1 was used as the source of annotated gene sets for GSEA. GSEA was conducted separately for each cell cluster of interest identified using Seurat.

### Statistical analysis

For single-cell sequencing analysis using Seurat, differential expression testing was based on the Wilcoxon rank-sum test (also known as the Mann–Whitney U test), a non-parametric test used to compare two independent samples (26).

For GSEA using fgsea, an enrichment score (ES) was calculated based on a vector of gene-level t-statistics from a differential expression test, with an empirical null distribution calculated by sampling random gene sets for each input pathway of interest. Then, a P-value was estimated by taking the number of random gene sets with the same or more extreme ES value divided by the total number of generated gene sets, and multiple hypothesis correction was applied, yielding adjusted P-values.

## Results

### Profiles of neutrophils, macrophages, B cells, and T cells in sepsis

**Figure 1:**
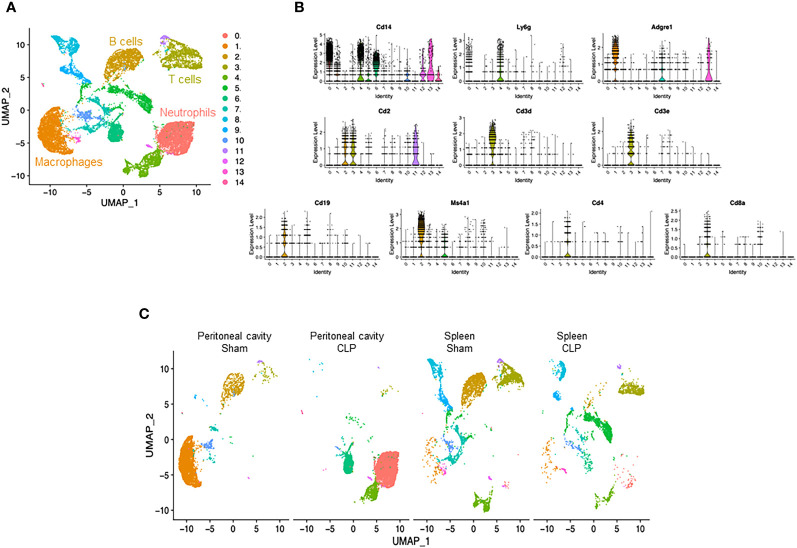
Identification of neutrophils, macrophages, B cells, and T cells. **(A)** UMAP plots showing the results of post-filtering unsupervised random forest classification of all groups combined. **(B)** Violin plots of key markers used for identifying cell types. **(C)** UMAP plots showing the post-filtering unsupervised random forest classification of each group (peritoneal cavity of sham and CLP, spleen of sham and CLP).

We induced sepsis in mice by CLP and isolated cells from the peritoneal cavity and spleen. The cells were then processed *via* the 10× Genomics scRNA-seq pipeline. After quality control (**Supplementary Figure 1**), we subjected the entire data set to unsupervised classification. This classification resulted in 14 clusters (#0–13), which were lined up in the order of cell numbers (**Figure 1A**, **Supplementary Figures 2**, **3**). Then, we sought to identify the cell types of the four major clusters using the putative markers *Cd14*, myeloid cells; *Cd19*, *Ms4a1*, B cells; and *Cd2*, *Cd3d/e*, *Cd4*, *Cd8a*, T cells (**Figure 1B**), along with the markers for other cell types (**Supplementary Figure 4**). Two major clusters were identified to be myeloid cells (#0, #1) (**Supplementary Figure 5**), and we further distinguished them by the markers for neutrophils (*Ly6g*) and macrophages (*Adgre1*). As such, each of the four major clusters was identified as follows: #0, neutrophils; #1, macrophages; #2, B cells; #3, T cells (**Figure 1A**). Next, we assessed the differences in cell distribution among the two compartments in sham and septic conditions. Neutrophils existed predominantly in the peritoneal cavity of septic mice and to a lesser extent in the spleen of sham and septic mice, whereas they were barely detectable in the peritoneal cavity of sham mice (**Figure 1C**). Macrophages existed predominantly in the peritoneal cavity of sham mice and to a lesser extent in the spleen of sham and septic mice, whereas they were barely detectable in the peritoneal cavity of septic mice (**Figure 1C**). B cells existed mainly in the peritoneal cavity and spleen of sham mice and to a lesser extent in the spleen of septic mice, whereas they were barely detectable in the peritoneal cavity of septic mice (**Figure 1C**). T cells were found mainly in the spleen of sham and septic mice and to a lesser extent in the peritoneal cavity of sham and septic mice (**Figure 1C**). Hence, variations in the presence of innate and adaptive immune cells across different compartments have been noted under both normal and septic conditions.

### Transcriptomic heterogeneity in neutrophils, macrophages, B cells, and T cells in sepsis

**Figure 2:**
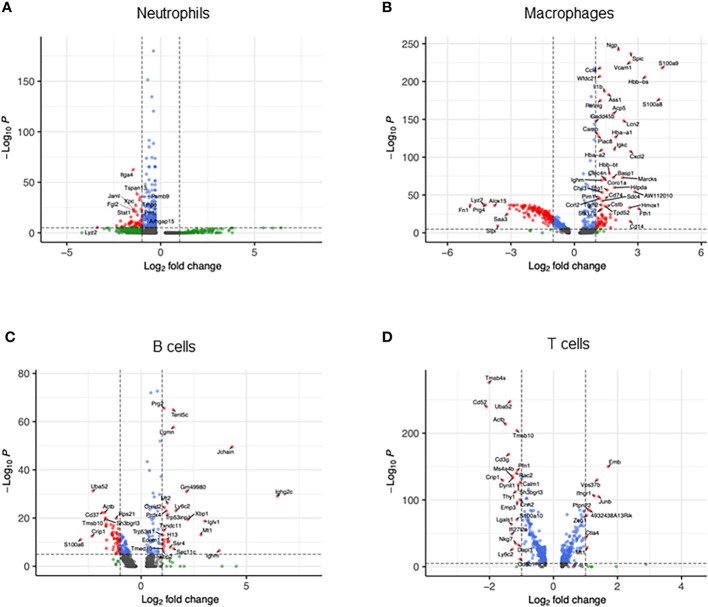
Transcriptomic heterogeneity caused by sepsis. Volcano plots showing the differentially expressed genes of CLP vs. sham in **(A)** neutrophils, **(B)** macrophages, **(C)** B cells, and **(D)** T cells.

We then compared transcriptomic differences caused by sepsis in each cell type by identifying differentially expressed genes and pathways in CLP mice compared with sham mice. In neutrophils, the most differentially expressed genes in septic mice compared with the control were *Cxcl2*, *Ccl4*, *CCl3*, *Il1rn*, *Fth1*, *Cxcl3*, *Thbs1*, *Acod1*, *Ier3*, and *Sod2* (**Figure 2A**, **Supplementary Table 1**). Based on these differentially expressed genes, pathways activated in the neutrophils of CLP mice were determined to be the ones related to inflammatory signaling such as NF-κB and responses to PAMPs, cytokines, and hypoxia (**Table 1**). In macrophages, the most differentially expressed genes after sepsis include *S100a9*, *S100a8*, *Hbb-bs*, *Fth1*, *AW112010*, *Spic*, *Cxcl2*, *Cd14*, *Hmox1*, and *Vcam1* (**Figure 2B**, **Supplementary Table 2**). Activated pathways in macrophages of septic mice were the ones related to cell aging, inflammatory signaling such as NF-κB, and responses to PAMPs (**Table 2**). In B cells, the most upregulated genes in septic mice were *Ighg2c*, *Jchain*, *Ighm*, *Iglv1*, *Mtq*, *Xbp1*, *Gm49980*, *Herpud1*, *Ly6c2*, and *Sec11c* (**Figure 2C**, **Supplementary Table 3**). Activated pathways relevant to B cells according to these genes were mostly the ones related to endoplasmic reticulum stress (**Table 3**). In T cells, the most differentially expressed genes in septic mice include *Cxcl2*, *Emb*, *Junb*, *Vps37b*, *Ifngr1*, *4932438A13Rik*, *Ptpn22*, *Mt1*, *Zeb1*, and *S100a8* (**Figure 2D**, **Supplementary Table 4**). The activated pathways in T cells of septic mice were the ones related to inflammatory signaling such as NF-κB, responses to PAMPs, and acute lung injury (**Table 4**). Overall, in sepsis different kinds of cells are activated and exhibited enhanced inflammatory signaling pathways in response to inflammatory mediators and circulatory failure.

**Table 1:** Differentially expressed pathways in septic neutrophils.

pathway	NES
HALLMARK_TNFA_SIGNALING_VIA_NFKB	3.26
GALINDO_IMMUNE_RESPONSE_TO_ENTEROTOXIN	3.09
SEKI_INFLAMMATORY_RESPONSE_LPS_UP	2.89
GROSS_HYPOXIA_VIA_ELK3_DN	2.86
NEMETH_INFLAMMATORY_RESPONSE_LPS_UP	2.83
GROSS_HYPOXIA_VIA_ELK3_AND_HIF1A_UP	2.63
GOBP_INFLAMMATORY_RESPONSE	2.57
HALLMARK_INFLAMMATORY_RESPONSE	2.49
GOBP_CELLULAR_RESPONSE_TO_INTERLEUKIN_1	2.49
GOMF_CYTOKINE_ACTIVITY	2.48
FOSTER_TOLERANT_MACROPHAGE_UP	2.45
REL_TARGET_GENES	2.45
GOBP_RESPONSE_TO_INTERLEUKIN_1	2.45
WP_LUNG_FIBROSIS	2.45
GOMF_CYTOKINE_RECEPTOR_BINDING	2.45
PLASARI_TGFB1_TARGETS_1HR_UP	2.43
TABULA_MURIS_SENIS_BLADDER_ENDOTHELIAL_ CELL_AGEING	2.41
RASHI_RESPONSE_TO_IONIZING_RADIATION_2	2.4
LIAN_LIPA_TARGETS_6M	2.4
GOMF_SIGNALING_RECEPTOR_REGULATOR_ACTIVITY	2.4
LIAN_LIPA_TARGETS_3M	2.4
GROSS_ELK3_TARGETS_DN	2.39
GROSS_HYPOXIA_VIA_ELK3_ONLY_UP	2.39
ZHENG_IL22_SIGNALING_UP	2.39
SAFFORD_T_LYMPHOCYTE_ANERGY	2.35
GOBP_PATTERN_RECOGNITION_RECEPTOR_ SIGNALING_PATHWAY	2.35
GERY_CEBP_TARGETS	2.34
GROSS_HYPOXIA_VIA_HIF1A_DN	2.34
RASHI_RESPONSE_TO_IONIZING_RADIATION_1	2.33
HALLMARK_HYPOXIA	2.33

NES, normalized enrichment score.

**Table 2:** Differentially expressed pathways in septic macrophages.

pathway	NES
TABULA_MURIS_SENIS_LIVER_MYELOID_LEUKOCYTE_AGEING	2.99
GALINDO_IMMUNE_RESPONSE_TO_ENTEROTOXIN	2.97
SEKI_INFLAMMATORY_RESPONSE_LPS_UP	2.75
TABULA_MURIS_SENIS_HEART_AND_AORTA_CARDIOMYOCYTE_AGEING	2.64
TABULA_MURIS_SENIS_MARROW_PRECURSOR_B_CELL_AGEING	2.56
TABULA_MURIS_SENIS_LIMB_MUSCLE_MACROPHAGE_AGEING	2.55
TABULA_MURIS_SENIS_GONADAL_ADIPOSE_TISSUE_MYELOID_CELL_AGEING	2.54
HALLMARK_TNFA_SIGNALING_VIA_NFKB	2.54
TABULA_MURIS_SENIS_BLADDER_ENDOTHELIAL_CELL_AGEING	2.46
TABULA_MURIS_SENIS_SPLEEN_CD8_POSITIVE_ALPHA_BETA_T_CELL_AGEING	2.45
RASHI_NFKB1_TARGETS	2.42
WP_LUNG_FIBROSIS	2.4
GOBP_PEPTIDYL_CYSTEINE_MODIFICATION	2.36
GOBP_RESPONSE_TO_HYDROGEN_PEROXIDE	2.36
TABULA_MURIS_SENIS_SUBCUTANEOUS_ADIPOSE_TISSUE_MYELOID_CELL_AGEING	2.36
TABULA_MURIS_SENIS_SPLEEN_T_CELL_AGEING	2.35
TABULA_MURIS_SENIS_BLADDER_BLADDER_UROTHELIAL_CELL_AGEING	2.33
HOUSTIS_ROS	2.33
GOBP_TRANSITION_METAL_ION_HOMEOSTASIS	2.33
GOBP_IRON_ION_HOMEOSTASIS	2.33
TABULA_MURIS_SENIS_KIDNEY_KIDNEY_DISTAL_CONVOLUTED_TUBULE_EPITHELIAL_CELL_AGEING	2.31
CHYLA_CBFA2T3_TARGETS_DN	2.31
TABULA_MURIS_SENIS_MARROW_HEMATOPOIETIC_PRECURSOR_CELL_AGEING	2.3
JIANG_AGING_HYPOTHALAMUS_UP	2.3
TABULA_MURIS_SENIS_LUNG_MATURE_NK_T_CELL_AGEING	2.3
GOBP_RESPONSE_TO_REACTIVE_OXYGEN_SPECIES	2.25
VILIMAS_NOTCH1_TARGETS_UP	2.24
TABULA_MURIS_SENIS_MARROW_MACROPHAGE_AGEING	2.24
TABULA_MURIS_SENIS_SPLEEN_CD4_POSITIVE_ALPHA_BETA_T_CELL_AGEING	2.23
GOBP_CELLULAR_TRANSITION_METAL_ION_HOMEOSTASIS	2.22

NES, normalized enrichment score.

**Table 3:** Differentially expressed pathways in septic B cells.

pathway	NES
PASQUALUCCI_LYMPHOMA_BY_GC_STAGE_UP	3.83
GOCC_ENDOPLASMIC_RETICULUM_PROTEIN_CONTAINING_COMPLEX	2.93
MORI_PLASMA_CELL_UP	2.92
LAZARO_GENETIC_MOUSE_MODEL_HIGH_GRADE_LARGE_CELL_NEUROENDOCRINE_LUNG_CARCINOMA_UP	2.68
REACTOME_BINDING_AND_UPTAKE_OF_LIGANDS_BY_SCAVENGER_RECEPTORS	2.67
GOBP_ENDOPLASMIC_RETICULUM_TO_CYTOSOL_TRANSPORT	2.66
VANASSE_BCL2_TARGETS_UP	2.57
GOBP_PROTEIN_EXIT_FROM_ENDOPLASMIC_RETICULUM	2.55
GOBP_RESPONSE_TO_ENDOPLASMIC_RETICULUM_STRESS	2.53
GOCC_NUCLEAR_OUTER_MEMBRANE_ENDOPLASMIC_RETICULUM_MEMBRANE_NETWORK	2.51
GOCC_ENDOPLASMIC_RETICULUM_CHAPERONE_COMPLEX	2.51
GOMF_IMMUNOGLOBULIN_RECEPTOR_BINDING	2.49
GOCC_IMMUNOGLOBULIN_COMPLEX	2.47
REACTOME_ROLE_OF_PHOSPHOLIPIDS_IN_PHAGOCYTOSIS	2.47
QI_PLASMACYTOMA_DN	2.46
GOBP_PHAGOCYTOSIS_RECOGNITION	2.45
GOCC_INTRINSIC_COMPONENT_OF_ENDOPLASMIC_RETICULUM_MEMBRANE	2.44
GOBP_ANTIGEN_PROCESSING_AND_PRESENTATION_OF_PEPTIDE_ANTIGEN_VIA_MHC_CLASS_IB	2.42
chr16A3	2.4
BURTON_ADIPOGENESIS_3	2.4
BOYLAN_MULTIPLE_MYELOMA_PCA3_DN	2.4
SHIN_B_CELL_LYMPHOMA_CLUSTER_2	2.4
GOCC_MHC_CLASS_I_PROTEIN_COMPLEX	2.39
GOBP_ANTIGEN_PROCESSING_AND_PRESENTATION_VIA_MHC_CLASS_IB	2.39
GOBP_ERAD_PATHWAY	2.37
GOBP_COMPLEMENT_ACTIVATION	2.35
chr8C5	2.34
GOCC_ENDOPLASMIC_RETICULUM_LUMEN	2.34
GOMF_ANTIGEN_BINDING	2.33
GOBP_DEFENSE_RESPONSE_TO_BACTERIUM	2.33

NES, normalized enrichment score.

**Table 4:** Differentially expressed pathways in septic T cells.

pathway	NES
HALLMARK_TNFA_SIGNALING_VIA_NFKB	2.7
GROSS_HYPOXIA_VIA_ELK3_DN	2.56
RASHI_RESPONSE_TO_IONIZING_RADIATION_2	2.55
GALINDO_IMMUNE_RESPONSE_TO_ENTEROTOXIN	2.51
SEKI_INFLAMMATORY_RESPONSE_LPS_UP	2.51
MCDOWELL_ACUTE_LUNG_INJURY_UP	2.42
YAO_TEMPORAL_RESPONSE_TO_PROGESTERONE_CLUSTER_1	2.37
CHIARADONNA_NEOPLASTIC_TRANSFORMATION_KRAS_CDC25_DN	2.34
GROSS_ELK3_TARGETS_DN	2.33
RASHI_NFKB1_TARGETS	2.28
MARSON_FOXP3_TARGETS_DN	2.27
VARELA_ZMPSTE24_TARGETS_UP	2.27
GOBP_CELLULAR_TRANSITION_METAL_ION_HOMEOSTASIS	2.26
GOBP_RESPONSE_TO_MOLECULE_OF_BACTERIAL_ORIGIN	2.23
GERY_CEBP_TARGETS	2.2
TABULA_MURIS_SENIS_HEART_AND_AORTA_SMOOTH_MUSCLE_CELL_AGEING	2.19
MA_MYELOID_DIFFERENTIATION_DN	2.19
ABBUD_LIF_SIGNALING_1_UP	2.17
TABULA_MURIS_SENIS_BLADDER_ENDOTHELIAL_CELL_AGEING	2.16
GOBP_TRANSITION_METAL_ION_HOMEOSTASIS	2.15
LIAN_LIPA_TARGETS_6M	2.14
GOBP_INFLAMMATORY_RESPONSE	2.13
GOBP_REGULATION_OF_NUCLEAR_TRANSCRIBED_MRNA_POLY_A_TAIL_SHORTENING	2.13
GOBP_CYTOPLASMIC_PATTERN_RECOGNITION_RECEPTOR_SIGNALING_PATHWAY	2.13
LIAN_LIPA_TARGETS_3M	2.13
GOBP_POSITIVE_REGULATION_OF_ERYTHROCYTE_DIFFERENTIATION	2.13
GOBP_IRON_ION_HOMEOSTASIS	2.12
ZHENG_FOXP3_TARGETS_IN_THYMUS_UP	2.12
CHYLA_CBFA2T3_TARGETS_DN	2.12
MIR_467A_3P	2.11

NES, normalized enrichment score.

### Neutrophil subset analysis

**Figure 3:**
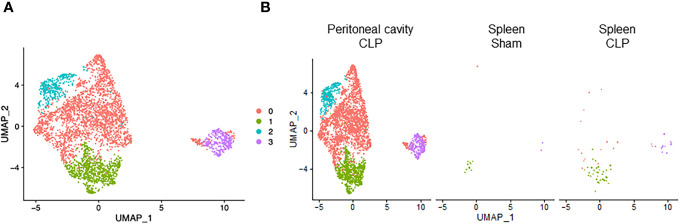
Distribution of neutrophil subsets. UMAP plots showing the subsets of neutrophils in **(A)** all groups combined and **(B)** each group.

To explore potential subsets within a cell type, we individually subjected each cell type to further classification. We found that neutrophils consisted of four clusters (**Figure 3A**). Cluster #0 was the major population and almost exclusively found in the peritoneal cavity of septic mice (**Figure 3B**), suggesting that this subtype plays a dominant role specifically at the site of infection. On the other hand, cluster #1 was found in both peritoneal cavity and spleen (**Figure 3B**), suggesting that this subset may migrate and localize in different compartments transiently. cluster #2 was almost exclusively found in the peritoneal cavity of septic mice similar to cluster #0 (**Figure 3B**). Cluster #3, the smallest population, was mostly found in the peritoneal cavity of septic mice but also to a lesser extent in the spleen of sham and septic mice (**Figure 3B**). Next, we listed the most differentially expressed genes for each cluster compared with the rest of neutrophils. In cluster #0, *Csf3*, *Chka*, *Hmox1*, *Hcar2*, *Camp*, *Cstb*, *Gde1*, *Gadd45g*, *Cpeb4*, and *Syne1* were the most upregulated genes (**Supplementary Table 5**). Positively differential pathways in this cluster were the ones related to iron uptake, transport and homeostasis, secondary lysosome and amino acid regulation by mammalian target of rapamycin complex 1 (mTORC1), and glycolysis and glucogenesis (**Table 5**). In cluster #1, the most upregulated genes were *Il1b*, *Pou2f2*, *Emp3*, *Ifitm1, Gm19951, Nr4a1, Pglyrp1, Prr13, S100a6*, and *Klf2* (**Supplementary Table 5**). The activated pathways were the ones related to cell aging, negative regulation of protein serine/threonine kinase, e.g., MAPK, and immunoregulatory cell–cell interaction (**Table 5**). In cluster #2, *Saa3, Orm1, Il10, Plin2, Csf3, Sod2, Lamp1, Gde1, Fnip2*, and *Cd63* were the most upregulated genes (**Supplementary Table 5**). The activated pathways in this cluster were the ones related to hematopoietic late progenitor cells, protein–lipid complex, plasminogen activation, inflammatory response, and fever generation (**Table 5**). In cluster #3, *Edn1*, *Rpsa*, *Rpl13*, *Spp1, Rpl32, Rps24, Rps20, Rps19, Rps8*, and *Rplp0* were the most upregulated genes (**Supplementary Table 5**), which reflected the activated pathways related to ribosome (**Table 5**). Taken together, neutrophils consist of different kinds of subsets, which include enhanced effector functions or immunoregulatory properties as well as altered cell metabolism.

**Table 5:** Differentially expressed pathways in neutrophil subsets.

CL	pathway	NES
0	REACTOME_IRON_UPTAKE_AND_TRANSPORT	2.29
0	GOCC_SECONDARY_LYSOSOME	2.24
0	GOBP_TRANSITION_METAL_ION_HOMEOSTASIS	2.23
0	REACTOME_AMINO_ACIDS_REGULATE_MTORC1	2.2
0	GOBP_CELLULAR_TRANSITION_METAL_ION_HOMEOSTASIS	2.2
0	GOBP_PEPTIDE_CROSS_LINKING	2.18
0	GOCC_PEPTIDASE_INHIBITOR_COMPLEX	2.18
0	MODY_HIPPOCAMPUS_POSTNATAL	2.13
0	WP_GLYCOLYSIS_AND_GLUCONEOGENESIS	2.12
0	GOCC_AUTOLYSOSOME	2.08
0	GOMF_CYSTEINE_TYPE_ENDOPEPTIDASE_INHIBITOR_ACTIVITY	2.06
0	ZFP617_TARGET_GENES	2.02
0	TABULA_MURIS_SENIS_BLADDER_ENDOTHELIAL_CELL_AGEING	2.01
0	GROSS_HYPOXIA_VIA_ELK3_DN	1.99
0	GOBP_NEGATIVE_REGULATION_OF_OXIDOREDUCTASE_ACTIVITY	1.87
0	REACTOME_TRANSPORT_OF_SMALL_MOLECULES	1.86
0	REACTOME_DOWNREGULATION_OF_SMAD2_3_SMAD4_TRANSCRIPTIONAL_ACTIVITY	1.86
0	REACTOME_PEXOPHAGY	1.85
0	GOBP_COLLATERAL_SPROUTING	1.84
0	GOBP_ETHANOLAMINE_CONTAINING_COMPOUND_METABOLIC_PROCESS	1.63
1	TABULA_MURIS_SENIS_SUBCUTANEOUS_ADIPOSE_TISSUE_MYELOID_CELL_AGEING	2.66
1	TABULA_MURIS_SENIS_MAMMARY_GLAND_STROMAL_CELL_AGEING	2.43
1	TABULA_MURIS_SENIS_MAMMARY_GLAND_MACROPHAGE_AGEING	2.42
1	LAZARO_GENETIC_MOUSE_MODEL_HIGH_GRADE_SMALL_CELL_NEUROENDOCRINE_LUNG_CARCINOMA_DN	2.35
1	TABULA_MURIS_SENIS_LUNG_B_CELL_AGEING	2.33
1	GOBP_NEGATIVE_REGULATION_OF_MAP_KINASE_ACTIVITY	2.32
1	BOYLAN_MULTIPLE_MYELOMA_D_DN	2.31
1	REACTOME_IMMUNOREGULATORY_INTERACTIONS_BETWEEN_A_LYMPHOID_AND_A_NON_LYMPHOID_CELL	2.28
1	GOCC_CYTOPLASMIC_SIDE_OF_MEMBRANE	2.26
1	TABULA_MURIS_SENIS_SPLEEN_CD4_POSITIVE_ALPHA_BETA_T_CELL_AGEING	2.2
1	GOBP_NEGATIVE_REGULATION_OF_PROTEIN_SERINE_THREONINE_KINASE_ACTIVITY	2.19
1	QI_PLASMACYTOMA_UP	2.17
1	GOBP_REGULATION_OF_INSULIN_RECEPTOR_SIGNALING_PATHWAY	2.16
1	GOMF_ACTIN_BINDING	2.16
1	GOCC_EXTRINSIC_COMPONENT_OF_CYTOPLASMIC_SIDE_OF_PLASMA_MEMBRANE	2.15
1	TORCHIA_TARGETS_OF_EWSR1_FLI1_FUSION_UP	2.13
1	GOBP_REGULATION_OF_PLATELET_ACTIVATION	2.13
1	GOCC_SIDE_OF_MEMBRANE	2.13
1	GOBP_REGULATION_OF_CELL_MORPHOGENESIS	2.13
1	GOBP_RESPONSE_TO_ORGANOPHOSPHORUS	2.12
2	IVANOVA_HEMATOPOIESIS_LATE_PROGENITOR	2.46
2	GOCC_PROTEIN_LIPID_COMPLEX	2.29
2	GOBP_PLASMINOGEN_ACTIVATION	2.05
2	REACTOME_SYNTHESIS_OF_15_EICOSATETRAENOIC_ACID_DERIVATIVES	-1.62
2	BIERIE_INFLAMMATORY_RESPONSE_TGFB1	-1.63
2	GOBP_POSITIVE_REGULATION_OF_FEVER_GENERATION	-1.63
2	GOBP_POSITIVE_REGULATION_OF_HEAT_GENERATION	-1.63
2	GOBP_REGULATION_OF_FEVER_GENERATION	-1.63
2	GOBP_REGULATION_OF_HEAT_GENERATION	-1.63
2	SHIN_B_CELL_LYMPHOMA_CLUSTER_6	-1.63
2	GOMF_NITRIC_OXIDE_SYNTHASE_REGULATOR_ACTIVITY	-1.82
2	GOBP_APOPTOTIC_PROCESS	-1.82
2	GOBP_NEGATIVE_REGULATION_OF_MOLECULAR_FUNCTION	-1.9
2	ELL2_TARGET_GENES	-1.91
2	LAZARO_GENETIC_MOUSE_MODEL_HIGH_GRADE_SMALL_CELL_NEUROENDOCRINE_LUNG_CARCINOMA_DN	-1.91
2	NFATC2_TARGET_GENES	-1.92
2	GOBP_RESPONSE_TO_INTERLEUKIN_17	-1.93
2	TABULA_MURIS_SENIS_LIVER_NK_CELL_AGEING	-1.97
2	GOBP_POSITIVE_REGULATION_OF_P38MAPK_CASCADE	-1.99
2	TABULA_MURIS_SENIS_LIMB_MUSCLE_T_CELL_AGEING	-2
3	GOCC_RIBOSOMAL_SUBUNIT	3.76
3	GOCC_RIBOSOME	3.75
3	GOMF_STRUCTURAL_CONSTITUENT_OF_RIBOSOME	3.75
3	GOCC_CYTOSOLIC_RIBOSOME	3.74
3	WP_CYTOPLASMIC_RIBOSOMAL_PROTEINS	3.72
3	GOMF_STRUCTURAL_MOLECULE_ACTIVITY	3.68
3	REACTOME_NONSENSE_MEDIATED_DECAY_NMD_INDEPENDENT_OF_THE_EXON_JUNCTION_COMPLEX_EJC	3.66
3	REACTOME_MAJOR_PATHWAY_OF_RRNA_PROCESSING_IN_THE_NUCLEOLUS_AND_CYTOSOL	3.64
3	GOBP_CYTOPLASMIC_TRANSLATION	3.64
3	REACTOME_SRP_DEPENDENT_COTRANSLATIONAL_PROTEIN_TARGETING_TO_MEMBRANE	3.63
3	REACTOME_NONSENSE_MEDIATED_DECAY_NMD	3.6
3	REACTOME_FORMATION_OF_A_POOL_OF_FREE_40S_SUBUNITS	3.59
3	REACTOME_EUKARYOTIC_TRANSLATION_INITIATION	3.58
3	REACTOME_TRANSLATION	3.54
3	GOCC_CYTOSOLIC_LARGE_RIBOSOMAL_SUBUNIT	3.43
3	GOCC_LARGE_RIBOSOMAL_SUBUNIT	3.41
3	TABULA_MURIS_SENIS_KIDNEY_EPITHELIAL_CELL_OF_PROXIMAL_TUBULE_AGEING	3.39
3	TABULA_MURIS_SENIS_THYMUS_PROFESSIONAL_ANTIGEN_PRESENTING_CELL_AGEING	3.38
3	TABULA_MURIS_SENIS_SPLEEN_NK_CELL_AGEING	3.34
3	BILANGES_SERUM_AND_RAPAMYCIN_SENSITIVE_GENES	3.33

CL, cluster; NES, normalized enrichment score.

### Macrophage subset analysis

**Figure 4:**
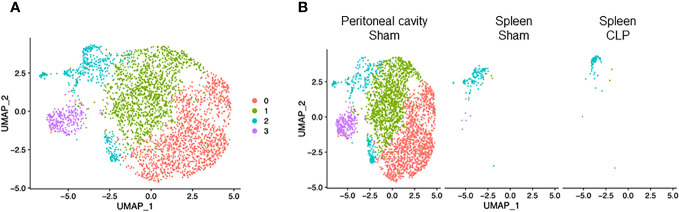
Distribution of macrophage subsets. UMAP plots showing the subsets of macrophages in **(A)** all groups combined and **(B)** each group.

Macrophages consisted of four clusters (**Figure 4A**). Two major clusters, cluster #0 and #1, were mostly found in the peritoneal cavity of sham mice (**Figure 4B**), suggesting they are bona fide peritoneal resident macrophages. Cluster #2 was the main population in the spleen of sham and CLP mice and also found in the peritoneal cavity of sham mice (**Figure 4B**), indicating that this population may have motility. Cluster #3, the smallest population, was mostly found in the peritoneal cavity of CLP mice (**Figure 4B**), suggesting they are specific to the site of infection. In cluster #0, the most upregulated genes were *Slpi*, *Chil3*, *S100a4*, *S100a6*, *Fabp5*, *Prtn3*, *Apoc2*, *Cd52*, *Tmem176b*, and *Fabp4* (**Supplementary Table 6**). Activated pathways in this cluster were signal-recognition particle (SPR)-dependent cotranslational protein targeting the membrane, ribosome, and cell aging (**Table 6**). In cluster #1, the most upregulated genes were *Cxcl13, Apoc1, Vsig4, Wnt2, Tgfb2, Timd4, C4b, Cald1, Scn1b*, and *Cdkn2a* (**Supplementary Table 6**). Activated pathways in this cluster were related to extracellular matrix organization, cytokine activity, coagulation, and cell adhesion mediated by integrin (**Table 6**). In cluster #2, the most upregulated genes were *S100a9, S100a8, Cd74, AW112010, Hmox1, Vcam1, Hbb-bs, Marcks, Spic*, and *Aif1* (**Supplementary Table 6**). Pathways activated in this cluster were cell aging and lymphocyte activation, adhesion, and proliferation (**Table 6**). In cluster #3, the most upregulated genes were *S100a9, S100a8, Cd74, AW112010, Hmox1, Vcam1, Hbb-bs, Marcks, Spic*, and *Aif1* (**Supplementary Table 6**). Pathways activated in this cluster were E2F and Myc targets (**Table 6**). Together, macrophage subsets exhibit different properties in cell aging, inflammatory activity, and mobility.

**Table 6:** Differentially expressed pathways in macrophage subsets.

CL	pathway	NES
0	REACTOME_SRP_DEPENDENT_COTRANSLATIONAL_PROTEIN_TARGETING_TO_MEMBRANE	3.48
0	WP_CYTOPLASMIC_RIBOSOMAL_PROTEINS	3.08
0	CHIARADONNA_NEOPLASTIC_TRANSFORMATION_KRAS_CDC25_DN	2.99
0	MCBRYAN_PUBERTAL_TGFB1_TARGETS_DN	2.97
0	TABULA_MURIS_SENIS_MARROW_MEGAKARYOCYTE_ERYTHROID_PROGENITOR_CELL_AGEING	2.96
0	GOCC_CYTOSOLIC_RIBOSOME	2.91
0	REACTOME_NONSENSE_MEDIATED_DECAY_NMD_INDEPENDENT_OF_THE_EXON_JUNCTION_COMPLEX_EJC	2.85
0	TABULA_MURIS_SENIS_MARROW_GRANULOCYTE_AGEING	2.85
0	TABULA_MURIS_SENIS_MARROW_GRANULOCYTOPOIETIC_CELL_AGEING	2.79
0	INGRAM_SHH_TARGETS_DN	2.75
0	GOMF_STRUCTURAL_CONSTITUENT_OF_RIBOSOME	2.73
0	GOCC_CYTOSOLIC_LARGE_RIBOSOMAL_SUBUNIT	2.71
0	HALLMARK_KRAS_SIGNALING_UP	2.69
0	BREDEMEYER_RAG_SIGNALING_NOT_VIA_ATM_DN	2.4
0	TABULA_MURIS_SENIS_MARROW_ERYTHROBLAST_AGEING	2.37
0	REACTOME_FORMATION_OF_FIBRIN_CLOT_CLOTTING_CASCADE	2.37
0	HARRIS_BRAIN_CANCER_PROGENITORS	2.35
0	REACTOME_TRIGLYCERIDE_METABOLISM	2.16
0	GOCC_CHYLOMICRON	2.1
0	BOYLAN_MULTIPLE_MYELOMA_C_D_DN	2.09
1	REACTOME_EXTRACELLULAR_MATRIX_ORGANIZATION	2.5
1	GOMF_CYTOKINE_ACTIVITY	2.5
1	HALLMARK_COAGULATION	2.5
1	MIR_5101	2.47
1	GOMF_HEPARIN_BINDING	2.44
1	GOBP_CELL_ADHESION_MEDIATED_BY_INTEGRIN	2.4
1	GOMF_CARGO_RECEPTOR_ACTIVITY	2.39
1	GOCC_COLLAGEN_CONTAINING_EXTRACELLULAR_MATRIX	2.38
1	GOMF_OPSONIN_BINDING	2.38
1	GOCC_EXTERNAL_ENCAPSULATING_STRUCTURE	2.35
1	GOMF_COMPLEMENT_BINDING	2.35
1	GOBP_REGULATION_OF_CELLULAR_RESPONSE_TO_GROWTH_FACTOR_STIMULUS	2.35
1	GOMF_G_PROTEIN_COUPLED_RECEPTOR_BINDING	2.3
1	REACTOME_ELASTIC_FIBRE_FORMATION	2.3
1	REACTOME_MOLECULES_ASSOCIATED_WITH_ELASTIC_FIBRES	2.3
1	LEE_BMP2_TARGETS_UP	2.29
1	CUI_TCF21_TARGETS_2_DN	2.29
1	AFFAR_YY1_TARGETS_UP	2.27
1	GOBP_INTEGRIN_ACTIVATION	2.26
1	GOBP_TRANSMEMBRANE_RECEPTOR_PROTEIN_SERINE_THREONINE_KINASE_SIGNALING_PATHWAY	2.25
2	TABULA_MURIS_SENIS_LIMB_MUSCLE_MACROPHAGE_AGEING	3.02
2	GOBP_POSITIVE_REGULATION_OF_LYMPHOCYTE_ACTIVATION	2.96
2	ICHIBA_GRAFT_VERSUS_HOST_DISEASE_35D_UP	2.91
2	GOBP_POSITIVE_REGULATION_OF_LEUKOCYTE_CELL_CELL_ADHESION	2.9
2	QI_PLASMACYTOMA_UP	2.88
2	WUNDER_INFLAMMATORY_RESPONSE_AND_CHOLESTEROL_UP	2.88
2	DESCARTES_ORGANOGENESIS_WHITE_BLOOD_CELLS	2.83
2	REACTOME_RHO_GTPASE_EFFECTORS	2.83
2	GOBP_POSITIVE_REGULATION_OF_LEUKOCYTE_PROLIFERATION	2.82
2	TABULA_MURIS_SENIS_SPLEEN_CD8_POSITIVE_ALPHA_BETA_T_CELL_AGEING	2.81
2	TABULA_MURIS_SENIS_SPLEEN_T_CELL_AGEING	2.81
2	LIAN_LIPA_TARGETS_6M	2.79
2	GOBP_LEUKOCYTE_CELL_CELL_ADHESION	2.78
2	LIAN_LIPA_TARGETS_3M	2.78
2	YU_MYC_TARGETS_DN	2.78
2	TABULA_MURIS_SENIS_LIVER_MYELOID_LEUKOCYTE_AGEING	2.77
2	TABULA_MURIS_SENIS_MARROW_HEMATOPOIETIC_PRECURSOR_CELL_AGEING	2.76
2	GOBP_ANTIGEN_PROCESSING_AND_PRESENTATION_OF_EXOGENOUS_PEPTIDE_ANTIGEN	2.76
2	GOBP_POSITIVE_REGULATION_OF_CELL_ACTIVATION	2.75
2	TABULA_MURIS_SENIS_MARROW_MONOCYTE_AGEING	2.72
3	HALLMARK_E2F_TARGETS	3.08
3	BURTON_ADIPOGENESIS_3	2.98
3	LAZARO_GENETIC_MOUSE_MODEL_HIGH_GRADE_LARGE_CELL_NEUROENDOCRINE_LUNG_CARCINOMA_UP	2.97
3	HALLMARK_MYC_TARGETS_V1	2.96
3	MARKEY_RB1_ACUTE_LOF_UP	2.93
3	MORI_IMMATURE_B_LYMPHOCYTE_DN	2.9
3	LAZARO_GENETIC_MOUSE_MODEL_HIGH_GRADE_SMALL_CELL_NEUROENDOCRINE_LUNG_CARCINOMA_UP	2.88
3	MORI_LARGE_PRE_BII_LYMPHOCYTE_UP	2.87
3	BERENJENO_TRANSFORMED_BY_RHOA_UP	2.82
3	MORI_PRE_BI_LYMPHOCYTE_UP	2.8
3	GOBERT_OLIGODENDROCYTE_DIFFERENTIATION_UP	2.79
3	HOFFMANN_LARGE_TO_SMALL_PRE_BII_LYMPHOCYTE_UP	2.79
3	HALLMARK_G2M_CHECKPOINT	2.73
3	KAMMINGA_EZH2_TARGETS	2.73
3	KONG_E2F3_TARGETS	2.72
3	ISHIDA_E2F_TARGETS	2.72
3	REACTOME_ACTIVATION_OF_THE_PRE_REPLICATIVE_COMPLEX	2.71
3	GOBP_DNA_REPLICATION	2.71
3	REACTOME_ACTIVATION_OF_ATR_IN_RESPONSE_TO_REPLICATION_STRESS	2.7
3	GOBP_DNA_TEMPLATED_DNA_REPLICATION	2.7

CL, cluster; NES, normalized enrichment score.

### B-cell subset analysis

**Figure 5:**
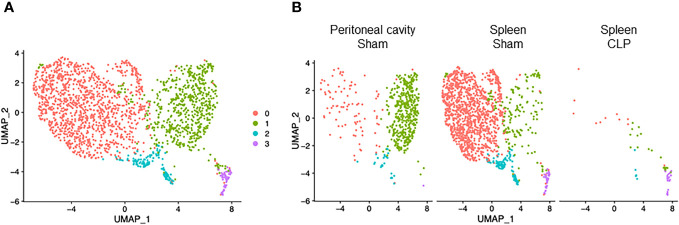
Distribution of B cell subsets. UMAP plots showing the subsets of B cells in **(A)** all groups combined and **(B)** each group.

B cells consisted of four clusters (**Figure 5A**). Cluster #0 was found mainly in the spleen of sham mice and to a lesser extent in the peritoneal cavity of sham and spleen of septic mice (**Figure 5B**). Cluster #1 was found mainly in the peritoneal cavity of sham mice and to a lesser extent in the spleen of sham and septic mice (**Figure 5B**). Clusters #2 and #3 consisted of a low number of cells and were found in three different samples (**Figure 5B**). B cells are known to be classified into B-1 and B-2 cells. Using the genes identified to be specific for either B-1 and B-2 cells in a previous study (27), clusters #0 and #2 aligned well with B-2 cells and cluster #1 aligned well with B-1 cells (**Supplementary Figure 6**). In cluster #0, the most upregulated genes were *Ighd*, *Ebf1*, *Mef2c*, *Fcer2a, Btg1, BE692007, H2-Aa, Vpreb3, H2-Ab1*, and *Cd69* (**Supplementary Table 7**). Pathways activated in this cluster were as follows: mature B lymphocyte, MHC protein complex, plasma cell, large pre-B-2 lymphocytes, protein complex binding (**Table 7**). In cluster #1, the most upregulated genes were *S100a6*, *Crip1*, *Vim*, *Ahnak*, *Plac8*, *S100a4*, *Iglc1, Lgals1, Tagln2*, and *Lyz2* (**Supplementary Table 7**). Activated pathways according to these genes were related to ribosomes and aging (**Table 7**). In cluster #2, the most upregulated genes were *Hmgb2, Ncl, Mif, Npm1, Hist1h1b, Eif5a, Ran, Nme1, Stmn1*, and *Pclaf* (**Supplementary Table 7**). Activated pathways in this cluster were mostly the ones related to cell aging (**Table 7**). In cluster #3, the most upregulated genes were *Jchain*, *Ighg2c, Jchain, Ighg2b, Igkc, Ighm, Slpi, Iglv1, Hsp90b1, Xbp1*, and *Iglc1* (**Supplementary Table 7**). Activated pathways in this cluster were related to the endoplasmic reticulum (**Table 7**). Taken together, B cells encompass both B-1 and B-2 cell types, with their respective subsets exhibiting distinct phenotypes indicative of cell maturation or aging. This represents the diverse developmental stages these cells undergo, playing crucial roles in both innate and adaptive immune functions during sepsis.

**Table 7:** Differentially expressed pathways in B cell subsets.

CL	pathway	NES
0	MORI_MATURE_B_LYMPHOCYTE_UP	3.41
0	MORI_PLASMA_CELL_DN	3.18
0	YU_MYC_TARGETS_DN	3.17
0	GOCC_MHC_PROTEIN_COMPLEX	3.16
0	GOMF_MHC_PROTEIN_COMPLEX_BINDING	2.86
0	MORI_LARGE_PRE_BII_LYMPHOCYTE_DN	2.86
0	GOCC_MHC_CLASS_II_PROTEIN_COMPLEX	2.8
0	GOMF_MHC_CLASS_II_PROTEIN_COMPLEX_BINDING	2.8
0	GOBP_ANTIGEN_PROCESSING_AND_PRESENTATION_OF_EXOGENOUS_PEPTIDE_ANTIGEN	2.67
0	GOBP_PEPTIDE_ANTIGEN_ASSEMBLY_WITH_MHC_CLASS_II_PROTEIN_COMPLEX	2.64
0	ZHENG_FOXP3_TARGETS_IN_T_LYMPHOCYTE_DN	2.58
0	GOBP_ANTIGEN_PROCESSING_AND_PRESENTATION_OF_EXOGENOUS_PEPTIDE_ANTIGEN_VIA_MHC_CLASS_II	2.57
0	GOBP_ANTIGEN_PROCESSING_AND_PRESENTATION_OF_EXOGENOUS_ANTIGEN	2.57
0	GOMF_PEPTIDE_ANTIGEN_BINDING	2.51
0	GOBP_REGULATION_OF_HUMORAL_IMMUNE_RESPONSE	2.48
0	GOBP_T_CELL_DIFFERENTIATION_IN_THYMUS	2.44
0	MIR_106A_3P_MIR_17_3P	2.42
0	MIR_20B_3P	2.42
0	PASQUALUCCI_LYMPHOMA_BY_GC_STAGE_DN	2.41
0	LAZARO_GENETIC_MOUSE_MODEL_HIGH_GRADE_LARGE_CELL_NEUROENDOCRINE_LUNG_CARCINOMA_DN	2.4
1	TABULA_MURIS_SENIS_SPLEEN_MATURE_NK_T_CELL_AGEING	2.93
1	WP_CYTOPLASMIC_RIBOSOMAL_PROTEINS	2.88
1	GOCC_CYTOSOLIC_RIBOSOME	2.85
1	TABULA_MURIS_SENIS_SUBCUTANEOUS_ADIPOSE_TISSUE_B_CELL_AGEING	2.84
1	TABULA_MURIS_SENIS_HEART_AND_AORTA_FIBROBLAST_OF_CARDIAC_TISSUE_AGEING	2.81
1	REACTOME_NONSENSE_MEDIATED_DECAY_NMD_INDEPENDENT_OF_THE_EXON_JUNCTION_COMPLEX_EJC	2.77
1	REACTOME_SRP_DEPENDENT_COTRANSLATIONAL_PROTEIN_TARGETING_TO_MEMBRANE	2.77
1	GOBP_CYTOPLASMIC_TRANSLATION	2.76
1	REACTOME_NONSENSE_MEDIATED_DECAY_NMD	2.74
1	REACTOME_FORMATION_OF_A_POOL_OF_FREE_40S_SUBUNITS	2.73
1	TABULA_MURIS_SENIS_HEART_AND_AORTA_LEUKOCYTE_AGEING	2.71
1	GOMF_STRUCTURAL_MOLECULE_ACTIVITY	2.66
1	TABULA_MURIS_SENIS_MARROW_ERYTHROBLAST_AGEING	2.66
1	BILANGES_SERUM_AND_RAPAMYCIN_SENSITIVE_GENES	2.65
1	REACTOME_EUKARYOTIC_TRANSLATION_INITIATION	2.63
1	GOCC_CYTOSOLIC_LARGE_RIBOSOMAL_SUBUNIT	2.62
1	GOMF_STRUCTURAL_CONSTITUENT_OF_RIBOSOME	2.61
1	TABULA_MURIS_SENIS_KIDNEY_MACROPHAGE_AGEING	2.6
1	TABULA_MURIS_SENIS_MAMMARY_GLAND_MACROPHAGE_AGEING	2.57
1	TABULA_MURIS_SENIS_KIDNEY_FIBROBLAST_AGEING	2.56
2	HALLMARK_MYC_TARGETS_V1	2.7
2	TABULA_MURIS_SENIS_THYMUS_THYMOCYTE_AGEING	2.61
2	TABULA_MURIS_SENIS_SPLEEN_PROERYTHROBLAST_AGEING	2.5
2	TABULA_MURIS_SENIS_TONGUE_BASAL_CELL_OF_EPIDERMIS_AGEING	2.48
2	TABULA_MURIS_SENIS_LUNG_CLASSICAL_MONOCYTE_AGEING	2.44
2	GOCC_MYELIN_SHEATH	2.42
2	KARLSSON_TGFB1_TARGETS_UP	2.41
2	GOBP_TELOMERE_MAINTENANCE_VIA_TELOMERE_LENGTHENING	2.38
2	GOBP_RNA_TEMPLATED_DNA_BIOSYNTHETIC_PROCESS	2.36
2	MORI_PRE_BI_LYMPHOCYTE_UP	2.36
2	MORI_LARGE_PRE_BII_LYMPHOCYTE_UP	2.34
2	MORI_IMMATURE_B_LYMPHOCYTE_DN	2.34
2	GOCC_PROTON_TRANSPORTING_ATP_SYNTHASE_COMPLEX	2.33
2	ISHIDA_E2F_TARGETS	2.3
2	GOMF_UNFOLDED_PROTEIN_BINDING	2.3
2	LE_EGR2_TARGETS_UP	2.3
2	GOCC_TELOMERASE_HOLOENZYME_COMPLEX	2.29
2	GOBP_REGULATION_OF_PROTEIN_LOCALIZATION_TO_CHROMOSOME_TELOMERIC_REGION	2.28
2	HALLMARK_E2F_TARGETS	2.28
2	BERENJENO_TRANSFORMED_BY_RHOA_UP	2.28
3	PASQUALUCCI_LYMPHOMA_BY_GC_STAGE_UP	3.29
3	GOCC_ENDOPLASMIC_RETICULUM_PROTEIN_CONTAINING_COMPLEX	2.91
3	GOCC_NUCLEAR_OUTER_MEMBRANE_ENDOPLASMIC_RETICULUM_MEMBRANE_NETWORK	2.86
3	GOBP_RESPONSE_TO_ENDOPLASMIC_RETICULUM_STRESS	2.8
3	GOCC_ORGANELLE_SUBCOMPARTMENT	2.76
3	MORI_PLASMA_CELL_UP	2.73
3	GOCC_ENDOPLASMIC_RETICULUM	2.53
3	GOBP_ERAD_PATHWAY	2.53
3	GOCC_INTRINSIC_COMPONENT_OF_ENDOPLASMIC_RETICULUM_MEMBRANE	2.51
3	GOBP_UBIQUITIN_DEPENDENT_ERAD_PATHWAY	2.47
3	BOYLAN_MULTIPLE_MYELOMA_C_D_DN	2.47
3	GOBP_PROTEIN_LOCALIZATION_TO_ENDOPLASMIC_RETICULUM	2.46
3	BOYLAN_MULTIPLE_MYELOMA_PCA1_UP	2.45
3	GOBP_RESPONSE_TO_TOPOLOGICALLY_INCORRECT_PROTEIN	2.43
3	GOBP_ESTABLISHMENT_OF_PROTEIN_LOCALIZATION_TO_ENDOPLASMIC_RETICULUM	2.43
3	QI_PLASMACYTOMA_DN	2.41
3	GOBP_ENDOPLASMIC_RETICULUM_TO_GOLGI_VESICLE_MEDIATED_TRANSPORT	2.39
3	GOCC_ENDOPLASMIC_RETICULUM_LUMEN	2.39
3	GOBP_GLYCOPROTEIN_METABOLIC_PROCESS	2.39
3	GOBP_RESPONSE_TO_UNFOLDED_PROTEIN	2.37

CL, cluster; NES, normalized enrichment score.

### T-cell subset analysis

**Figure 6:**
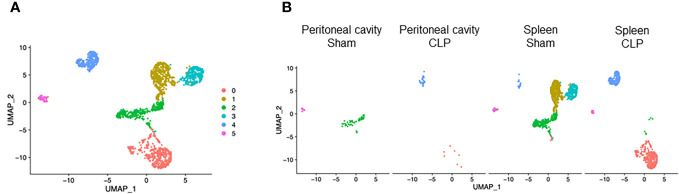
Distribution of T-cell subsets. UMAP plots showing the subsets of T cells in **(A)** all groups combined and **(B)** each group.

T cells consisted of six clusters (**Figure 6A**). Cluster #0 was found almost exclusively in the spleen of CLP mice and to a lesser extent in the peritoneal cavity of CLP mice (**Figure 6B**). On the other hand, clusters #2 and #3 were exclusive to the spleen of sham mice (**Figure 6B**). Cluster #1 was found mainly in the spleen of sham mice and also existed in the peritoneal cavity of sham mice and to a lesser extent in the spleen of CLP mice (**Figure 6B**). Cluster #4 was found mainly in the spleen of CLP mice and to a lesser extent in the spleen of sham mice and peritoneal cavity of CLP mice (**Figure 6B**). Cluster #5 formed a small population in the spleen of sham and CLP mice and the peritoneal cavity of sham mice (**Figure 6B**). Considering the high expressions of *Cd8b1* and *Cd8a* (**Supplementary Table 8**), cluster #3 seemed to be CD8 T cells. In cluster #0, the most upregulated genes were *Emb*, *Vps37b*, *Ifngr1*, *Junb, Fam241a, Ptpn22, Gramd3, Klhl6, 4932438A13Rikm*, and *Zeb1* (**Supplementary Table 8**). The pathways revealed that ribosome, cytoplasmic translation, and serum and rapamycin sensitive genes were upregulated (**Table 8**). In cluster #1, the most upregulated genes were *Igfbp4, Tspan32, Actb, Uba52, Cd52, Ly6c1, Tmsb4x, Ifi27l2a, Trbc2*, and *Limd2* (**Supplementary Table 8**). Activated pathways in this cluster were related to ribosome- and SRP-dependent co-translational protein targeting to the membrane (**Table 8**). In cluster #2, the most upregulated genes were *Ccl5, S100a6, Ly6c2, S100a4, Lgals1, Nkg7, AW112010, S100a10, Crip1*, and *Ahnak* (**Supplementary Table 8**). Activated pathways in this cluster were mostly related to cell aging (**Table 8**). In cluster #3, the most upregulated genes were *Cd8b1*, Cd8a, *Dapl1*, *Ccr9, Arl4c, Nkg7, Uba52, Tubb5, Cd52*, and *Dnajc15* (**Supplementary Table 8**). Activated pathways in this cluster were related to ribosome and structural molecule activity (**Table 8**). In cluster #4, the most upregulated genes were *Cxcl2*, *Lcn2*, *Fth1*, *Ccl4*, *Slpi*, *Ccl3*, *Neat1*, *Fcer1g*, *Ifitm2*, and *Mt1* (**Supplementary Table 8**). Activated pathways in this cluster were related to hypoxia, cell aging, and TNF-α signaling *via* NF-κB (**Table 8**). In cluster #5, the most upregulated genes were *Cd74*, *Igkc*, *H2-Aa*, *Cd79a*, *H2-Eb1*, *H2-Ab1*, *Ighm*, *Ly6d*, *Iglc2*, and *Ebf1* (**Supplementary Table 8**). Activated pathways in this cluster were mostly irrelevant to T cells (**Table 8**), suggesting that the population was too small to be classified properly. Together, T cells subsets have distinct characteristics in molecular translocation or cell activation.

**Table 8:** Differentially expressed pathways in T cell subsets.

CL	pathway	NES
0	WP_CYTOPLASMIC_RIBOSOMAL_PROTEINS	3.2
0	GOBP_CYTOPLASMIC_TRANSLATION	2.99
0	BILANGES_SERUM_AND_RAPAMYCIN_SENSITIVE_GENES	2.97
0	GOCC_CYTOSOLIC_RIBOSOME	2.9
0	GOMF_RRNA_BINDING	2.86
0	REACTOME_NONSENSE_MEDIATED_DECAY_NMD_INDEPENDENT_OF_THE_EXON_JUNCTION_COMPLEX_EJC	2.81
0	REACTOME_SRP_DEPENDENT_COTRANSLATIONAL_PROTEIN_TARGETING_TO_MEMBRANE	2.8
0	REACTOME_EUKARYOTIC_TRANSLATION_INITIATION	2.75
0	REACTOME_FORMATION_OF_A_POOL_OF_FREE_40S_SUBUNITS	2.75
0	REACTOME_NONSENSE_MEDIATED_DECAY_NMD	2.74
0	REACTOME_MAJOR_PATHWAY_OF_RRNA_PROCESSING_IN_THE_NUCLEOLUS_AND_CYTOSOL	2.66
0	TABULA_MURIS_SENIS_HEART_AND_AORTA_LEUKOCYTE_AGEING	2.63
0	BILANGES_SERUM_RESPONSE_TRANSLATION	2.58
0	GOMF_STRUCTURAL_CONSTITUENT_OF_RIBOSOME	2.53
0	GOBP_RIBOSOME_ASSEMBLY	2.46
0	TABULA_MURIS_SENIS_KIDNEY_FIBROBLAST_AGEING	2.45
0	GOCC_RIBOSOMAL_SUBUNIT	2.45
0	GOCC_RIBOSOME	2.45
0	GOBP_CYTOPLASMIC_PATTERN_RECOGNITION_RECEPTOR_SIGNALING_PATHWAY	2.38
0	TABULA_MURIS_SENIS_BLADDER_BLADDER_CELL_AGEING	2.35
1	GOCC_CYTOSOLIC_RIBOSOME	3.53
1	WP_CYTOPLASMIC_RIBOSOMAL_PROTEINS	3.44
1	GOCC_CYTOSOLIC_LARGE_RIBOSOMAL_SUBUNIT	3.35
1	REACTOME_SRP_DEPENDENT_COTRANSLATIONAL_PROTEIN_TARGETING_TO_MEMBRANE	3.35
1	REACTOME_FORMATION_OF_A_POOL_OF_FREE_40S_SUBUNITS	3.26
1	REACTOME_NONSENSE_MEDIATED_DECAY_NMD_INDEPENDENT_OF_THE_EXON_JUNCTION_COMPLEX_EJC	3.24
1	GOMF_STRUCTURAL_CONSTITUENT_OF_RIBOSOME	3.1
1	REACTOME_EUKARYOTIC_TRANSLATION_INITIATION	3.09
1	GOCC_RIBOSOMAL_SUBUNIT	3.03
1	REACTOME_NONSENSE_MEDIATED_DECAY_NMD	3.02
1	GOMF_STRUCTURAL_MOLECULE_ACTIVITY	3
1	GOCC_CYTOSOLIC_SMALL_RIBOSOMAL_SUBUNIT	2.95
1	REACTOME_MAJOR_PATHWAY_OF_RRNA_PROCESSING_IN_THE_NUCLEOLUS_AND_CYTOSOL	2.84
1	GOBP_CYTOPLASMIC_TRANSLATION	2.81
1	GOCC_RIBOSOME	2.81
1	GOCC_ACTIN_FILAMENT_BUNDLE	2.8
1	GOCC_LARGE_RIBOSOMAL_SUBUNIT	2.75
1	TABULA_MURIS_SENIS_DIAPHRAGM_SKELETAL_MUSCLE_SATELLITE_CELL_AGEING	2.7
1	GOCC_SMALL_RIBOSOMAL_SUBUNIT	2.68
1	HSF2_TARGET_GENES	2.65
2	TABULA_MURIS_SENIS_MAMMARY_GLAND_T_CELL_AGEING	3.05
2	TABULA_MURIS_SENIS_LUNG_CD4_POSITIVE_ALPHA_BETA_T_CELL_AGEING	2.92
2	TABULA_MURIS_SENIS_SPLEEN_CD8_POSITIVE_ALPHA_BETA_T_CELL_AGEING	2.79
2	TABULA_MURIS_SENIS_LUNG_CD8_POSITIVE_ALPHA_BETA_T_CELL_AGEING	2.68
2	TABULA_MURIS_SENIS_MAMMARY_GLAND_B_CELL_AGEING	2.67
2	TABULA_MURIS_SENIS_MAMMARY_GLAND_MACROPHAGE_AGEING	2.65
2	TABULA_MURIS_SENIS_BROWN_ADIPOSE_TISSUE_T_CELL_AGEING	2.65
2	LI_INDUCED_T_TO_NATURAL_KILLER_UP	2.61
2	TABULA_MURIS_SENIS_SPLEEN_T_CELL_AGEING	2.6
2	TABULA_MURIS_SENIS_MARROW_HEMATOPOIETIC_PRECURSOR_CELL_AGEING	2.55
2	TABULA_MURIS_SENIS_LUNG_B_CELL_AGEING	2.51
2	TABULA_MURIS_SENIS_THYMUS_DN4_THYMOCYTE_AGEING	2.49
2	TABULA_MURIS_SENIS_SUBCUTANEOUS_ADIPOSE_TISSUE_B_CELL_AGEING	2.45
2	TABULA_MURIS_SENIS_KIDNEY_MACROPHAGE_AGEING	2.45
2	TABULA_MURIS_SENIS_KIDNEY_T_CELL_AGEING	2.45
2	TABULA_MURIS_SENIS_GONADAL_ADIPOSE_TISSUE_B_CELL_AGEING	2.45
2	GOBP_NATURAL_KILLER_CELL_ACTIVATION	2.44
2	GOLDRATH_ANTIGEN_RESPONSE	2.44
2	GOCC_COLLAGEN_CONTAINING_EXTRACELLULAR_MATRIX	2.41
2	GOCC_EXTERNAL_ENCAPSULATING_STRUCTURE	2.4
3	GOCC_CYTOSOLIC_RIBOSOME	3.62
3	WP_CYTOPLASMIC_RIBOSOMAL_PROTEINS	3.58
3	GOMF_STRUCTURAL_CONSTITUENT_OF_RIBOSOME	3.56
3	GOCC_RIBOSOMAL_SUBUNIT	3.47
3	GOCC_RIBOSOME	3.45
3	GOMF_STRUCTURAL_MOLECULE_ACTIVITY	3.44
3	REACTOME_FORMATION_OF_A_POOL_OF_FREE_40S_SUBUNITS	3.44
3	REACTOME_NONSENSE_MEDIATED_DECAY_NMD_INDEPENDENT_OF_THE_EXON_JUNCTION_COMPLEX_EJC	3.39
3	REACTOME_SRP_DEPENDENT_COTRANSLATIONAL_PROTEIN_TARGETING_TO_MEMBRANE	3.38
3	REACTOME_MAJOR_PATHWAY_OF_RRNA_PROCESSING_IN_THE_NUCLEOLUS_AND_CYTOSOL	3.38
3	REACTOME_EUKARYOTIC_TRANSLATION_INITIATION	3.37
3	GOBP_CYTOPLASMIC_TRANSLATION	3.31
3	REACTOME_TRANSLATION	3.31
3	GOCC_CYTOSOLIC_LARGE_RIBOSOMAL_SUBUNIT	3.3
3	REACTOME_NONSENSE_MEDIATED_DECAY_NMD	3.28
3	GOCC_LARGE_RIBOSOMAL_SUBUNIT	3.26
3	BILANGES_SERUM_AND_RAPAMYCIN_SENSITIVE_GENES	3.01
3	GOCC_CYTOSOLIC_SMALL_RIBOSOMAL_SUBUNIT	2.83
3	GOCC_POLYSOMAL_RIBOSOME	2.82
3	GOCC_SMALL_RIBOSOMAL_SUBUNIT	2.81
4	GROSS_HYPOXIA_VIA_ELK3_DN	2.82
4	RASHI_RESPONSE_TO_IONIZING_RADIATION_2	2.66
4	TABULA_MURIS_SENIS_MARROW_GRANULOCYTOPOIETIC_CELL_AGEING	2.64
4	LIAN_LIPA_TARGETS_6M	2.59
4	LIAN_LIPA_TARGETS_3M	2.58
4	TABULA_MURIS_SENIS_PANCREAS_PANCREATIC_BETA_CELL_AGEING	2.57
4	HALLMARK_TNFA_SIGNALING_VIA_NFKB	2.56
4	GERY_CEBP_TARGETS	2.55
4	HESS_TARGETS_OF_HOXA9_AND_MEIS1_DN	2.52
4	TABULA_MURIS_SENIS_SPLEEN_MACROPHAGE_AGEING	2.51
4	GOBP_INFLAMMATORY_RESPONSE	2.5
4	GOBP_RESPONSE_TO_MOLECULE_OF_BACTERIAL_ORIGIN	2.49
4	TABULA_MURIS_SENIS_MAMMARY_GLAND_BASAL_CELL_AGEING	2.49
4	GALINDO_IMMUNE_RESPONSE_TO_ENTEROTOXIN	2.49
4	TABULA_MURIS_SENIS_SUBCUTANEOUS_ADIPOSE_TISSUE_MYELOID_CELL_AGEING	2.48
4	SEKI_INFLAMMATORY_RESPONSE_LPS_UP	2.48
4	CHYLA_CBFA2T3_TARGETS_DN	2.47
4	MIR_467A_3P	2.47
4	MIR_669B_3P	2.45
4	MIR_669F_3P	2.45
5	HOFFMANN_SMALL_PRE_BII_TO_IMMATURE_B_LYMPHOCYTE_UP	3.02
5	GOBP_B_CELL_ACTIVATION	3
5	GOBP_B_CELL_RECEPTOR_SIGNALING_PATHWAY	2.98
5	GOBP_B_CELL_MEDIATED_IMMUNITY	2.96
5	MORI_MATURE_B_LYMPHOCYTE_UP	2.92
5	MORI_LARGE_PRE_BII_LYMPHOCYTE_DN	2.92
5	GOBP_B_CELL_DIFFERENTIATION	2.91
5	YU_MYC_TARGETS_DN	2.89
5	GOBP_REGULATION_OF_B_CELL_ACTIVATION	2.89
5	TABULA_MURIS_SENIS_KIDNEY_FENESTRATED_CELL_AGEING	2.89
5	TABULA_MURIS_SENIS_LUNG_NON_CLASSICAL_MONOCYTE_AGEING	2.87
5	GOBP_REGULATION_OF_B_CELL_PROLIFERATION	2.86
5	GOBP_B_CELL_PROLIFERATION	2.84
5	MORI_IMMATURE_B_LYMPHOCYTE_UP	2.77
5	GOBP_ANTIGEN_PROCESSING_AND_PRESENTATION_OF_EXOGENOUS_ANTIGEN	2.76
5	GOBP_ANTIGEN_PROCESSING_AND_PRESENTATION_OF_PEPTIDE_ANTIGEN	2.76
5	GOBP_ANTIGEN_PROCESSING_AND_PRESENTATION	2.76
5	GOBP_ANTIGEN_PROCESSING_AND_PRESENTATION_OF_EXOGENOUS_PEPTIDE_ANTIGEN	2.75
5	GOBP_ANTIGEN_PROCESSING_AND_PRESENTATION_OF_PEPTIDE_OR_POLYSACCHARIDE_ANTIGEN_VIA_MHC_CLASS_II	2.73
5	TABULA_MURIS_SENIS_THYMUS_PROFESSIONAL_ANTIGEN_PRESENTING_CELL_AGEING	2.72

CL, cluster; NES, normalized enrichment score.

## Discussion

In this study, we have explored the transcriptomic profiling of immune cells including neutrophils, macrophages, B cells, and T cells in intra-abdominal sepsis using scRNA-seq, recognizing the pivotal roles these cells play in the host’s defense against pathogens. We have unveiled the frequency, phenotype, and subsets of those cell populations in the peritoneal cavity and spleen. While millions of cells were conveniently isolated from the peritoneal cavity of each mouse, the processing capacity for scRNA-seq using the 10x Genomics kit was limited to a maximum of 10,000. Consequently, the UMAPs generated reflect the proportion of the processed cells rather than the absolute cell counts. Notably, in CLP-induced sepsis mice, neutrophils emerged as the predominant population within the peritoneal cavity, thereby causing other cell types to appear relatively few in the UMAP (in terms of percentage of whole-cell population). We chose spleen as it is one of the most important lymphoid organs for the immune system. Moreover, circulating cells pass through or are trapped within the spleen; thus, splenocytes reflect the blood cells to a certain extent. Peritoneal cavity was chosen as the site of infection, where robust innate immune activities can be observed in response to the pathogens. We did not include PBMCs as they barely contain neutrophils, which are arguably the most important cell type during sepsis (3). Whole blood leukocytes, which contain neutrophils, would be worth evaluating but could be fragile for lengthy red blood cell lysis.

It has been traditionally thought that sepsis consists of an acute pro-inflammatory (hyperdynamic) phase and a later immunosuppressive (hypodynamic) state. This could still hold true in terms of the overall immune balance, but it is now known that both pro- and anti-inflammatory mediators and cells coexist from the early stage of sepsis (28). Thus, it is important to evaluate the status of cells not in bulk but individually to precisely understand their roles and potential interaction with other cells during sepsis. In fact, our scRNA-seq analysis revealed that the most cell types exhibited pro-inflammatory phenotypes such as increased gene levels of chemokines and DAMPs, including *Cxcl2*, *Ccl4*, *CCl3*, *S100a9*, and *S100a8*, and NF-κB activation in septic mice, whereas many of the subsets within the cell type were not characterized by the typical pro-inflammatory genes or pathways. Considering the fact that previously proposed sepsis drugs, which target the canonical pro-inflammatory mediators or signaling such as PAMPs or PRRs, have failed in the clinical trials (29), a more focused cell-specific approach might be needed to break through the current situation.

In the present study, we have identified multiple subsets within each major cell population in an unbiased way. Differentially expressed genes indicated that subsets were distinct from others in different ways, such as enhanced effector functions, immunoregulatory responses, cellular metabolism, and aging. The further assessments of phenotypical changes in different environments for individual clusters would enable us to define the characteristics of those subsets more clearly. It may also be interesting to investigate whether those subsets play any roles in other disease conditions. In fact, M1/M2 macrophages, which were initially studied extensively in oncology (30), are now known to contribute to sepsis and other disorders as well (31). The subsets in the present study were statistically defined in an unbiased way based on their gene expressions. Nevertheless, it is important to validate their significance in a focused approach. Isolation of specific subsets by cell sorting and studying them *in vitro* or adoptively transferring them *in vivo* would certify their impacts on the diseases more directly and strongly.

All major cell populations we analyzed here contained at least one subset possessing cellular aging properties. Cellular aging is regulated by metabolic and epigenetic changes and has a close relation with cell death mechanisms, both of which can be reflected by transcriptomics (32). We have previously shown that aged neutrophils significantly contributed to the development of sepsis. eCIRP, the major DAMP in sepsis, inhibited neutrophil apoptosis to induce aged neutrophils (10). Neutrophils possessing both aged and antigen-presenting properties induced Th1 differentiation to aggravate acute lung injury and worsen survival in sepsis (9). Moreover, this novel neutrophil subset was also identified in septic patients (9). In addition to neutrophils, however, cellular aging has not been studied extensively in most of other immune cell types such as macrophages and lymphocytes especially in sepsis. Our study implies the need for investigating cellular aging in different populations to better understand sepsis pathophysiology. Cellular aging can occur in response to cellular stress or damage irrespective of animals’ age (9, 10). Mice used in our study were in the same age for each group; thus, the difference in cellular aging status was unlikely to be due to the age of the animals. Nevertheless, it would be worth investigating whether cellular aging can be influenced by the age of the animals during sepsis in the future studies. It is also meaningful to evaluate the molecules and pathways related to cell death simultaneously to grasp the mechanisms of cellular aging from a wider perspective. Our previous studies revealed several novel subtypes of neutrophils, including APANs, nectin-2, and serpin B2 neutrophils, which were found to be upregulated during sepsis (9, 10, 33). However, our latest scRNA-seq data could not distinctly demonstrate these specific neutrophil subtypes. This discrepancy might be attributed to the limited resolution in the clustering process and the relatively low occurrence of these cell types in our current dataset.

Each cell population also contained subpopulations expressing the genes related to ribosomes, the organelles that facilitate protein synthesis from mRNA (34). Ribosomes are composed of ribosomal RNA, such as 18S (35), and ribosomal proteins (RP), such as RPSA, RPL13, and RPS24 (36). The roles of ribosomes are better known in viral rather than bacterial infection since host ribosomes are crucial for the expression of viral proteins (37). Our data suggest that ribosomes could be an interesting focus to be investigated in bacterial sepsis too. Ribosomal activities may be enhanced in sepsis with the demand for producing inflammatory molecules against infection or compensating lost proteins due to cellular damage. This system needs to be tightly regulated to avoid aberrant immune responses, which can cause tissue injury. It would also be of significance to combine the transcriptomics with proteomics to evaluate ribosomal activities since mRNA is translated to proteins by ribosomes (34).

To induce sepsis, we subjected mice to CLP, which is arguably the most-used animal model of sepsis. CLP depicts intraabdominal polymicrobial sepsis, a severe pathological condition with an extremely poor prognosis (38). Nevertheless, it has to be carefully considered when interpreting preclinical findings of sepsis and applying it in patients since sepsis is a heterogenetic disorder that can be caused in different ways. In addition to the polymicrobial model of sepsis, studying *E. coli* or endotoxemia or in some sterile injury model of sepsis can be informative to compare and contrast the gene expression profile among various model systems and conclude which model may mimic more closely with human sepsis in terms of transcriptional landscape (38). In this study, we analyzed samples from two compartments, peritoneal cavity and spleen. Since sepsis is accompanied by injury in multiple organs, including, but not limited to, lungs, liver, kidneys, and central nervous system (1), it would be of interest to further analyze our data along with the transcriptome of those different organs.

We studied the transcriptomic profile of immune cells in sepsis using the samples collected at 20 h after CLP. This specific time point was selected based on our extensive research in this area (5, 9, 13, 39). At that time point of CLP-induced sepsis, we have observed significant immune responses characterized by changes in leukocyte count, increased activation of immune cells, increased release of pro-inflammatory cytokines, and the elevation of organ injury markers in blood (5, 9, 13, 39). Conducting analyses at various time points is crucial; however, it often yields complex datasets that can be challenging to analyze comprehensively and conclusively correlate. Our data offer a valuable foundation for generating hypotheses and further exploration. Further studies on the dynamics of specific genes/pathways of interest as selected from our scRNA-seq data could enhance our understanding of disease severity. This approach would enable the correlation of gene expression profiles with systemic inflammation and organ function, thereby providing a more comprehensive understanding of the intricate dynamics underlying sepsis progression.

We acknowledge the limitations of our study. First, we collected the samples only at one time point as aforementioned; therefore, some of the cell populations were barely found in certain samples. This could also be the reason why some prominent pathways that are well known to be activated in sepsis, such as NLRP3 inflammasome pathway (40), were not significantly altered in our data. Additional evaluation at earlier and later time points would precisely depict the kinetics of the cell frequency and phenotype. Second, only observational data were included in the present study and the interventional studies were not conducted on the genes or cells we mentioned here. Knockdown or specific depletion of those genes or cells would firmly demonstrate the significance of our findings. Third, our paper does not contain human data. Recently, more and more scRNA-seq data have been available in the public database (41). Nevertheless, in addition to blood samples, it is still challenging to obtain patient samples from closed compartments or organs, such as peritoneal cavity or spleen, especially during sepsis. Incorporating patient data from these regions would contribute valuable insights and enhance the clinical relevance of animal studies.

In summary, we have comprehensively evaluated the distribution, phenotype, and subsets of immune cells under normal and septic conditions by implementing scRNA-seq. The frequency and characteristics of cells altered dramatically after sepsis in different compartments, and each cell population consisted of distinct subtypes. We have identified novel areas within sepsis that remain largely unexplored, notably encompassing cell aging across diverse cell types and ribosomal activities. These less traveled scientific areas present an enticing opportunity for future investigations. To further substantiate our current findings, there is a need for more targeted interventional studies concentrating on specific molecules and cells. The eagerly anticipated results from such studies will add rigor to and validate our present discoveries.

## Data availability statement

The datasets presented in this study can be found in online repositories. The names of the repository/repositories and accession number(s) can be found below: GSE249975 (GEO).

## Ethics statement

The animal study was approved by Feinstein Institutes for Medical Research. The study was conducted in accordance with the local legislation and institutional requirements.

## Author contributions

AM: Conceptualization, Data curation, Formal Analysis, Investigation, Methodology, Software, Validation, Visualization, Writing – original draft, Writing – review & editing. AJ: Conceptualization, Formal Analysis, Investigation, Methodology, Software, Writing – review & editing. MA: Conceptualization, Formal Analysis, Funding acquisition, Investigation, Methodology, Project administration, Resources, Supervision, Validation, Visualization, Writing – original draft, Writing – review & editing. PW: Conceptualization, Formal Analysis, Funding acquisition, Project administration, Resources, Supervision, Writing – review & editing.
