# Supplementary material for: Transcriptomic profiling of immune cells in murine polymicrobial sepsis
Source: Front Immunol. 2024 Jan 26;15:1347453. doi: 10.3389/fimmu.2024.1347453 (PMC10853340; doi:10.3389/fimmu.2024.1347453)
Supplement: Supplementary file 1 [file DataSheet_1.pdf]

Supplemental Figure 1. Quality control metrics.

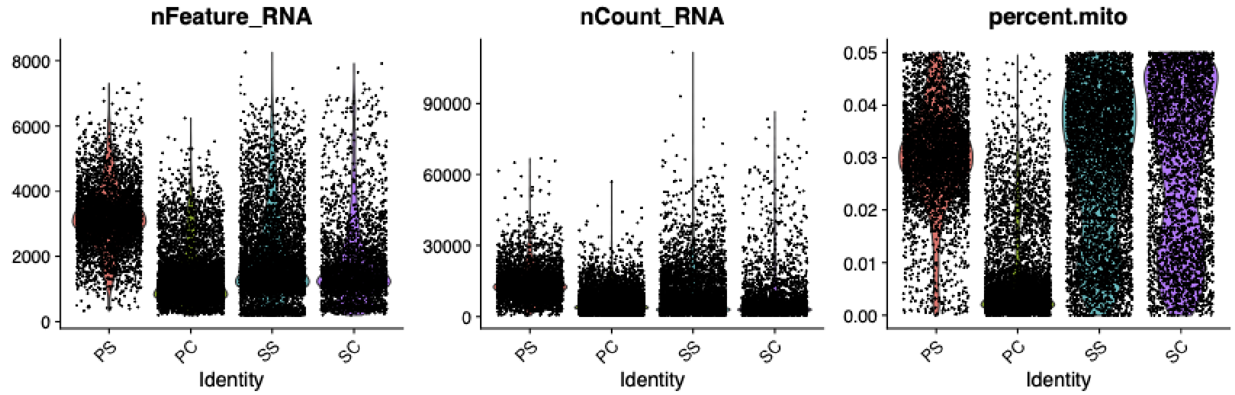

Plots showing the number of genes detected in each cell (nFeature_RNA), the number of molecules detected in each cell (nCount_RNA), and percent mitochondrial genes (percent.mito) after using DoubletFinder and applying cutoffs. PS, peritoneal cavity sham; PC, peritoneal cavity CLP; SS, spleen sham; SC, spleen CLP.

Supplemental Figure 2. Non-harmonized clusters.

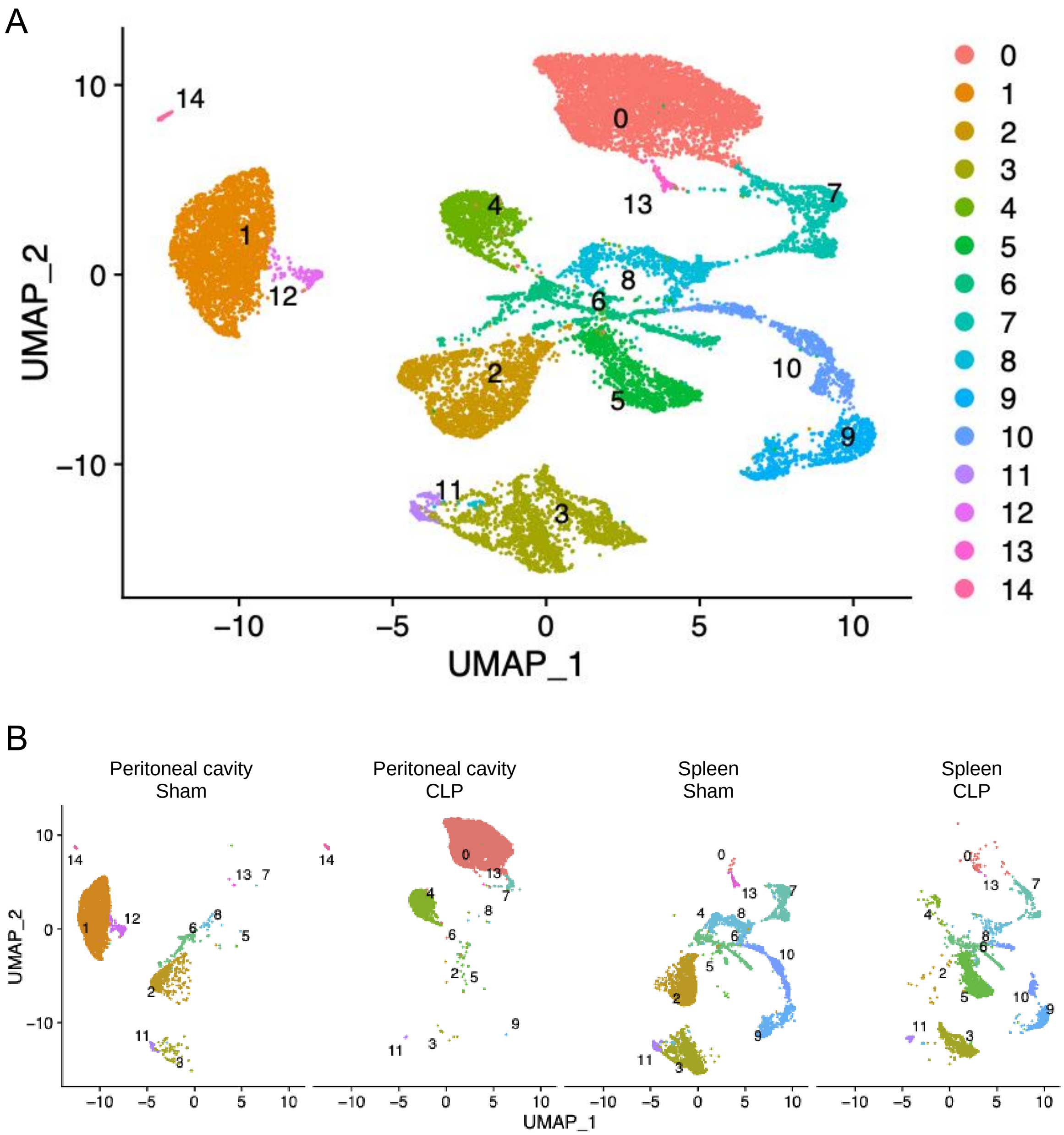

Non-harmonized UMAP plots for **(A)** all groups combined and **(B)** each group.

Supplemental Figure 3. Top expressed genes for all clusters.

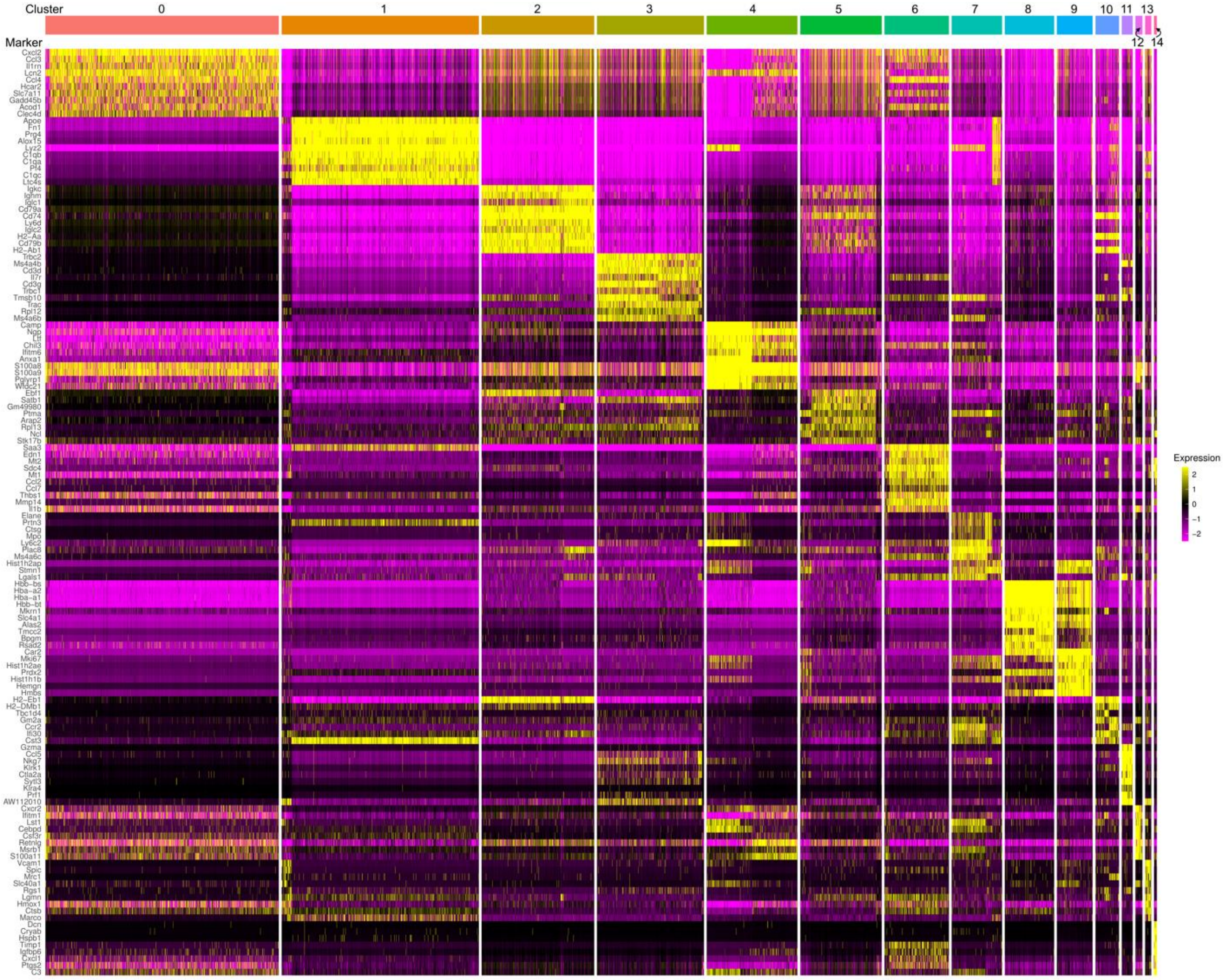

A heatmap showing the top expressed genes for all 14 clusters.

Supplemental Figure 4. Additional markers for identifying cell types.

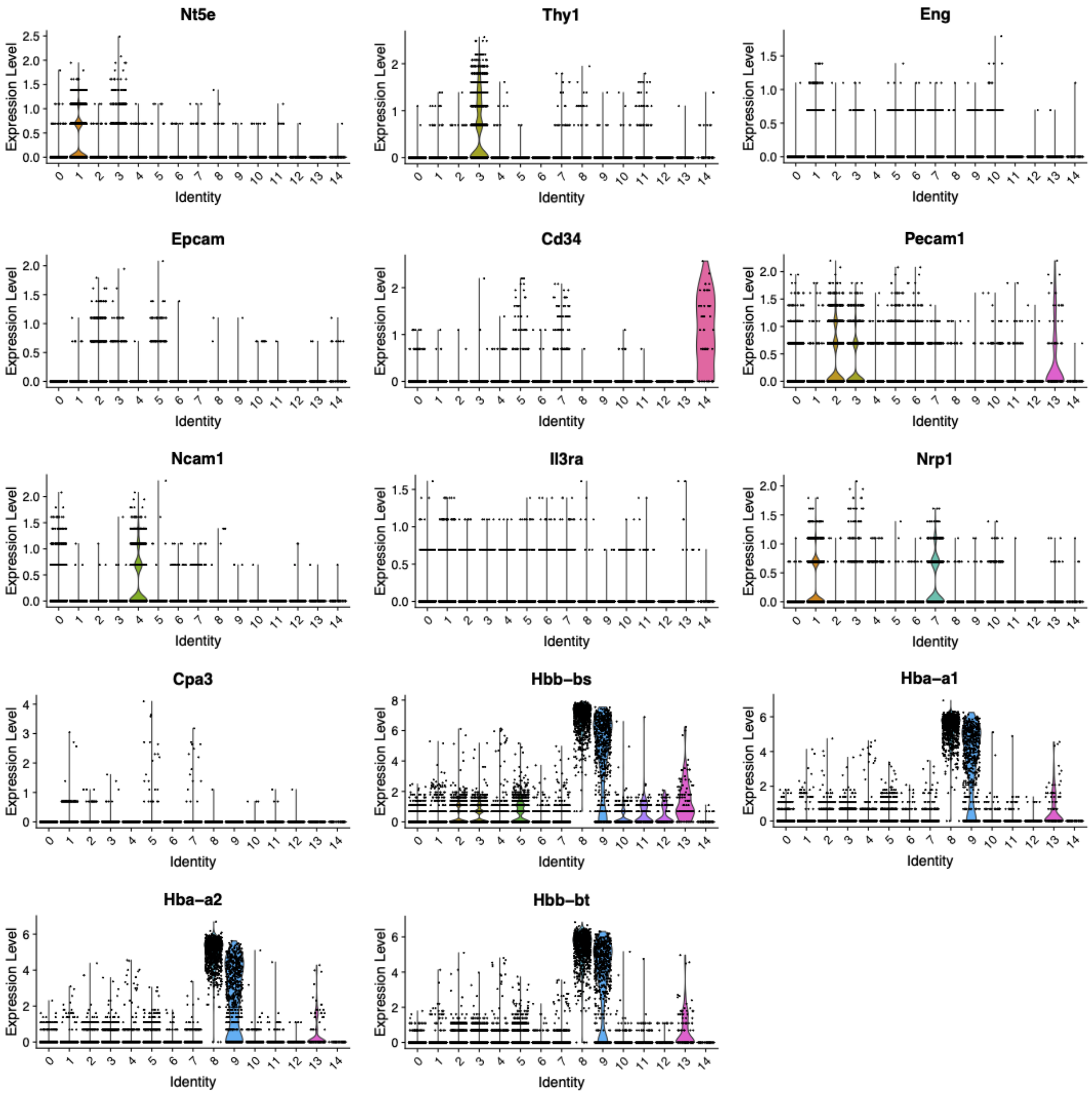

Violin plots of key markers for stromal cells (Nt5e, Thy1, Eng), epithelial cells (Epcam), endothelial-related cells (Cd34, Pecam1), natural killer cells (Ncam1), plasmacytoid dendritic cells (Il3ra, Nrp1), granulocytes (Cpa3), and erythroid cells (Hbb-bs, Hba-a2, Hba-a1, Hbb-bt).

Supplemental Figure 5. Initial annotations for all clusters.

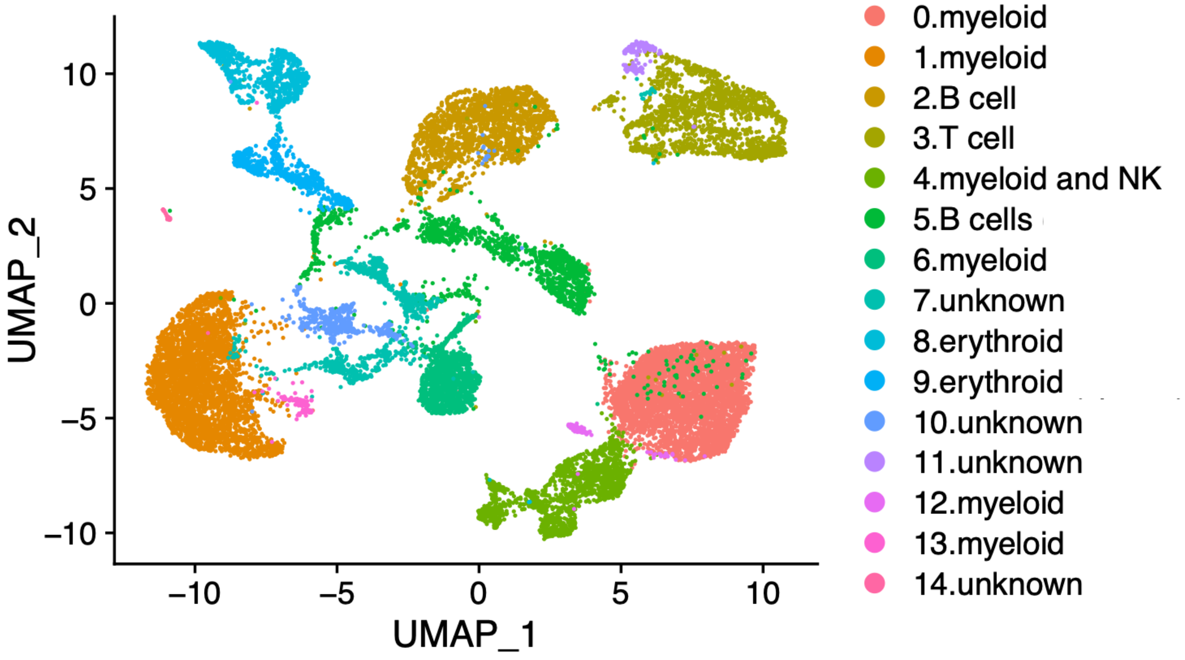

UMAP plots (all samples combined) with initial annotations for all clusters.

Supplemental Figure 6. Markers for B-1 and B-2 cells.

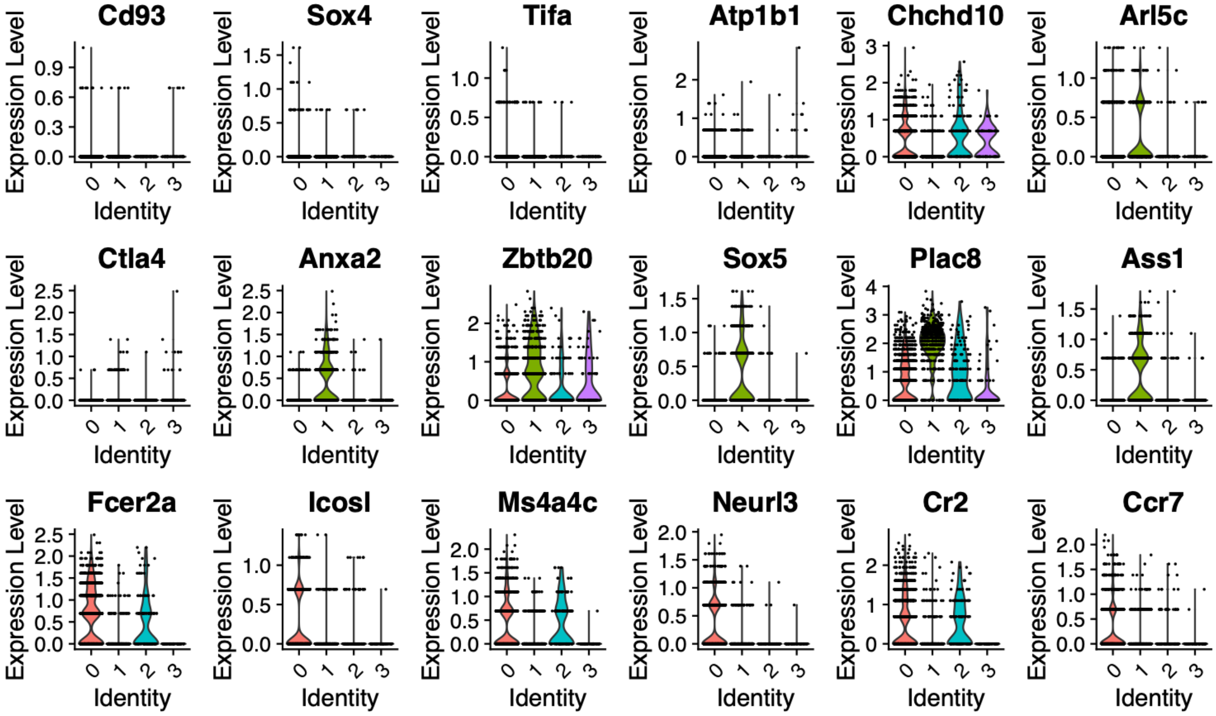

Violin plots of key markers used for identifying B-1 (cluster#1) and B-2 (cluster#0, #2) cells.
