# Supplementary material for: Transcriptomic profiling of immune cells in murine polymicrobial sepsis
Source: Front Immunol. 2024 Jan 26;15:1347453. doi: 10.3389/fimmu.2024.1347453 (PMC10853340; doi:10.3389/fimmu.2024.1347453)
Supplement: Supplementary file 2 [file Table_1.docx]

| **gene** | **p_val** | **avg_log2FC** | **pct.1** | **pct.2** | **p_val_adj** |
| --- | --- | --- | --- | --- | --- |
| Cxcl2 | 5.79E-10 | 6.413280372 | 0.999 | 0.692 | 1.06E-05 |
| Ccl4 | 1.02E-09 | 5.965637129 | 0.991 | 0.462 | 1.86E-05 |
| Ccl3 | 6.30E-10 | 5.459712332 | 0.997 | 0.462 | 1.15E-05 |
| Il1rn | 9.75E-10 | 3.844333271 | 0.996 | 0.538 | 1.78E-05 |
| Fth1 | 8.52E-10 | 3.775212429 | 1 | 1 | 1.56E-05 |
| Cxcl3 | 3.66E-08 | 3.661069444 | 0.895 | 0.077 | 0.000669383 |
| Thbs1 | 5.00E-07 | 3.63052419 | 0.9 | 0.538 | 0.009155499 |
| Acod1 | 7.76E-09 | 3.301941531 | 0.975 | 0.385 | 0.000142142 |
| Ier3 | 6.88E-09 | 3.174738877 | 0.971 | 0.154 | 0.000125911 |
| Sod2 | 1.38E-08 | 3.007454523 | 0.971 | 0.538 | 0.000252831 |
| Cd14 | 1.01E-08 | 2.848518981 | 0.991 | 0.615 | 0.000185437 |
| Slc15a3 | 3.32E-09 | 2.789347215 | 0.963 | 0.154 | 6.07E-05 |
| AA467197 | 1.91E-07 | 2.774931645 | 0.902 | 0.231 | 0.003504812 |
| Hcar2 | 1.12E-07 | 2.764043428 | 0.933 | 0.462 | 0.002058594 |
| Cstb | 3.06E-07 | 2.668610152 | 0.891 | 0.231 | 0.005607078 |
| Esd | 3.41E-08 | 2.63314833 | 0.946 | 0.538 | 0.000624168 |
| Slc7a11 | 3.55E-08 | 2.606391725 | 0.991 | 0.385 | 0.000649476 |
| Gadd45b | 1.39E-06 | 2.588243031 | 0.92 | 0.538 | 0.025370053 |
| Traf1 | 1.41E-07 | 2.551703117 | 0.84 | 0 | 0.002573076 |
| Lcn2 | 3.27E-09 | 2.52144503 | 1 | 0.846 | 5.99E-05 |
| Plin2 | 2.07E-07 | 2.387717377 | 0.922 | 0.385 | 0.003791864 |
| Furin | 3.22E-07 | 2.374205199 | 0.899 | 0.308 | 0.005890354 |
| Qsox1 | 3.76E-07 | 2.352423133 | 0.809 | 0 | 0.006885519 |
| Dock10 | 5.92E-07 | 2.314246655 | 0.845 | 0.154 | 0.010831692 |
| Pik3ap1 | 2.49E-08 | 2.272473704 | 0.924 | 0.154 | 0.000456237 |
| Nfkbia | 7.03E-08 | 2.211148442 | 0.998 | 0.923 | 0.00128672 |
| Tgoln1 | 3.34E-07 | 2.17238979 | 0.871 | 0.154 | 0.00611929 |
| Ctsb | 1.06E-07 | 2.136645661 | 0.948 | 0.692 | 0.001947127 |
| Sqstm1 | 1.71E-06 | 2.085459655 | 0.862 | 0.308 | 0.031357843 |
| Cd9 | 6.28E-08 | 1.950730067 | 0.981 | 0.769 | 0.001149681 |

**Supplemental Table 1. Differentially-expressed genes of CLP vs sham in neutrophils.**

avg_log2FC, log fold-change of the average expression between the two groups (positive values indicate that the gene is more highly expressed in CLP); pct.1, percent of CLP cells with detected expression of the gene; pct.2, percent of sham cells with detected expression the gene; p_val_adj, adjusted p-value based on Bonferroni correction using all genes in the dataset.

**Supplemental Table 2. Differentially-expressed genes of CLP vs sham in macrophages.**

| **gene** | **p_val** | **avg_log2FC** | **pct.1** | **pct.2** | **p_val_adj** |
| --- | --- | --- | --- | --- | --- |
| S100a9 | 4.75E-223 | 4.14810227 | 0.985 | 0.043 | 8.69E-219 |
| S100a8 | 2.02E-181 | 3.998803516 | 1 | 0.06 | 3.69E-177 |
| Hbb-bs | 5.45E-211 | 3.331775881 | 0.806 | 0.029 | 9.97E-207 |
| Fth1 | 6.15E-37 | 3.04187224 | 1 | 1 | 1.13E-32 |
| AW112010 | 3.10E-59 | 2.854162734 | 0.776 | 0.145 | 5.67E-55 |
| Spic | 5.57E-242 | 2.666825742 | 0.642 | 0.012 | 1.02E-237 |
| Cxcl2 | 1.31E-112 | 2.655776213 | 0.97 | 0.104 | 2.40E-108 |
| Cd14 | 4.84E-20 | 2.639939682 | 0.97 | 0.754 | 8.86E-16 |
| Hmox1 | 3.05E-38 | 2.6087444 | 0.896 | 0.309 | 5.58E-34 |
| Vcam1 | 5.02E-230 | 2.599418484 | 0.687 | 0.016 | 9.19E-226 |
| Lcn2 | 6.67E-153 | 2.335314649 | 0.955 | 0.066 | 1.22E-148 |
| Marcks | 6.50E-78 | 2.278322506 | 0.881 | 0.127 | 1.19E-73 |
| Ngp | 4.53E-247 | 2.082786354 | 0.955 | 0.035 | 8.29E-243 |
| Mt1 | 6.48E-22 | 1.951818661 | 1 | 0.884 | 1.19E-17 |
| Hba-a1 | 1.16E-131 | 1.94780814 | 0.313 | 0.005 | 2.13E-127 |
| Hilpda | 7.45E-65 | 1.891065628 | 0.403 | 0.028 | 1.36E-60 |
| Igkc | 3.40E-115 | 1.889392292 | 0.851 | 0.074 | 6.22E-111 |
| Acp5 | 6.43E-163 | 1.855732555 | 0.537 | 0.015 | 1.18E-158 |
| Basp1 | 4.58E-78 | 1.839633815 | 0.507 | 0.037 | 8.38E-74 |
| Lgmn | 5.71E-12 | 1.71372524 | 0.672 | 0.58 | 1.05E-07 |
| Tmsb10 | 2.02E-29 | 1.697402478 | 1 | 0.556 | 3.69E-25 |
| Rgs1 | 3.54E-25 | 1.695769287 | 0.507 | 0.127 | 6.47E-21 |
| Hbb-bt | 2.04E-83 | 1.652847543 | 0.254 | 0.006 | 3.74E-79 |
| Ass1 | 1.40E-187 | 1.618812693 | 0.478 | 0.008 | 2.57E-183 |
| Cstb | 1.40E-40 | 1.598705532 | 0.925 | 0.273 | 2.57E-36 |
| Btg1 | 1.47E-61 | 1.546316573 | 0.985 | 0.199 | 2.69E-57 |
| H3f3b | 3.56E-19 | 1.50027037 | 1 | 0.997 | 6.52E-15 |
| Gde1 | 8.22E-25 | 1.49763963 | 0.493 | 0.124 | 1.50E-20 |
| Cd47 | 7.97E-23 | 1.492045177 | 0.97 | 0.616 | 1.46E-18 |
| Cd74 | 5.44E-51 | 1.490674905 | 0.97 | 0.205 | 9.96E-47 |

avg_log2FC, log fold-change of the average expression between the two groups (positive values indicate that the gene is more highly expressed in CLP); pct.1, percent of CLP cells with detected expression of the gene; pct.2, percent of sham cells with detected expression the gene; p_val_adj, adjusted p-value based on Bonferroni correction using all genes in the dataset.

**Supplemental Table 3. Differentially-expressed genes of CLP vs sham in B cells.**

| **gene** | **p_val** | **avg_log2FC** | **pct.1** | **pct.2** | **p_val_adj** |
| --- | --- | --- | --- | --- | --- |
| Ighg2c | 4.33E-34 | 6.471125092 | 0.161 | 0.005 | 7.92E-30 |
| Jchain | 1.53E-54 | 4.324386159 | 0.571 | 0.054 | 2.81E-50 |
| Ighm | 1.74E-11 | 3.707038291 | 0.911 | 0.991 | 3.18E-07 |
| Iglv1 | 9.22E-24 | 3.01798691 | 0.5 | 0.097 | 1.69E-19 |
| Mt1 | 4.42E-18 | 2.842315803 | 0.446 | 0.107 | 8.09E-14 |
| Xbp1 | 2.22E-24 | 2.214844735 | 0.625 | 0.166 | 4.06E-20 |
| Gm49980 | 5.04E-36 | 2.158652631 | 0.911 | 0.317 | 9.23E-32 |
| Herpud1 | 4.96E-09 | 1.812147606 | 0.661 | 0.348 | 9.08E-05 |
| Ly6c2 | 4.61E-28 | 1.648488568 | 0.429 | 0.06 | 8.44E-24 |
| Sec11c | 2.54E-12 | 1.545415258 | 0.857 | 0.605 | 4.65E-08 |
| Lgmn | 1.07E-62 | 1.537886264 | 0.875 | 0.125 | 1.96E-58 |
| Tent5c | 6.37E-70 | 1.520091435 | 0.554 | 0.036 | 1.17E-65 |
| Mzb1 | 1.46E-06 | 1.489528043 | 0.911 | 0.785 | 0.026780827 |
| Ssr4 | 7.17E-13 | 1.380946872 | 0.893 | 0.626 | 1.31E-08 |
| Iglc2 | 4.60E-07 | 1.368291538 | 0.732 | 0.913 | 0.008419106 |
| Txndc5 | 5.57E-08 | 1.338450551 | 0.536 | 0.274 | 0.001019565 |
| Pim1 | 1.72E-15 | 1.315005231 | 0.75 | 0.329 | 3.15E-11 |
| B2m | 4.92E-15 | 1.26071944 | 1 | 0.961 | 9.01E-11 |
| Trp53inp1 | 1.61E-26 | 1.258549901 | 0.571 | 0.115 | 2.95E-22 |
| Mt2 | 2.72E-31 | 1.240922992 | 0.179 | 0.007 | 4.98E-27 |
| H13 | 3.44E-16 | 1.231325546 | 0.625 | 0.214 | 6.29E-12 |
| Prdx4 | 5.42E-28 | 1.201025257 | 0.589 | 0.119 | 9.93E-24 |
| Creld2 | 2.25E-29 | 1.099661888 | 0.518 | 0.085 | 4.13E-25 |
| Prg2 | 1.52E-70 | 1.094053974 | 0.357 | 0.011 | 2.79E-66 |
| Fkbp11 | 1.89E-13 | 1.090989797 | 0.357 | 0.083 | 3.47E-09 |
| Txndc11 | 6.17E-20 | 1.077305726 | 0.5 | 0.115 | 1.13E-15 |
| Edem1 | 5.10E-16 | 1.077011629 | 0.482 | 0.126 | 9.34E-12 |
| Mki67 | 6.39E-15 | 1.066782351 | 0.268 | 0.042 | 1.17E-10 |
| Tmed10 | 2.50E-12 | 1.048283084 | 0.804 | 0.413 | 4.58E-08 |
| Fkbp2 | 2.31E-11 | 1.046961581 | 0.518 | 0.201 | 4.22E-07 |

avg_log2FC, log fold-change of the average expression between the two groups (positive values indicate that the gene is more highly expressed in CLP); pct.1, percent of CLP cells with detected expression of the gene; pct.2, percent of sham cells with detected expression the gene; p_val_adj, adjusted p-value based on Bonferroni correction using all genes in the dataset.

**Supplemental Table 4. Differentially-expressed genes of CLP vs sham in T cells.**

| **gene** | **p_val** | **avg_log2FC** | **pct.1** | **pct.2** | **p_val_adj** |
| --- | --- | --- | --- | --- | --- |
| Cxcl2 | 1.80E-08 | 2.885948834 | 0.219 | 0.131 | 0.000330098 |
| Emb | 5.55E-155 | 1.710724432 | 0.899 | 0.668 | 1.02E-150 |
| Junb | 5.47E-110 | 1.411692764 | 0.821 | 0.403 | 1.00E-105 |
| Vps37b | 5.85E-135 | 1.359681849 | 0.788 | 0.298 | 1.07E-130 |
| Ifngr1 | 5.07E-111 | 1.27317505 | 0.845 | 0.44 | 9.27E-107 |
| 4932438A13Rik | 6.56E-89 | 1.144663106 | 0.746 | 0.377 | 1.20E-84 |
| Ptpn22 | 2.60E-90 | 1.089866822 | 0.797 | 0.466 | 4.75E-86 |
| Mt1 | 8.54E-34 | 1.048969906 | 0.211 | 0.038 | 1.56E-29 |
| Zeb1 | 9.59E-82 | 1.033813269 | 0.642 | 0.262 | 1.76E-77 |
| S100a8 | 3.52E-08 | 1.024677175 | 0.847 | 0.897 | 0.000643854 |
| Ctla4 | 4.64E-49 | 1.008914347 | 0.304 | 0.058 | 8.49E-45 |
| Ramp3 | 8.68E-88 | 0.953686484 | 0.367 | 0.027 | 1.59E-83 |
| Socs3 | 5.44E-80 | 0.950760212 | 0.569 | 0.175 | 9.95E-76 |
| Cblb | 1.52E-88 | 0.947654136 | 0.649 | 0.238 | 2.78E-84 |
| Srgn | 2.01E-35 | 0.939286413 | 0.911 | 0.791 | 3.68E-31 |
| Gramd3 | 6.36E-66 | 0.937971215 | 0.706 | 0.385 | 1.16E-61 |
| Klhl6 | 6.64E-48 | 0.925304876 | 0.626 | 0.359 | 1.22E-43 |
| Txnip | 2.68E-45 | 0.917291296 | 0.866 | 0.768 | 4.91E-41 |
| Bcl2l11 | 9.83E-79 | 0.883043781 | 0.547 | 0.161 | 1.80E-74 |
| Ssh2 | 1.13E-70 | 0.87952687 | 0.806 | 0.493 | 2.07E-66 |
| Tnfrsf4 | 3.17E-08 | 0.87698911 | 0.167 | 0.087 | 0.000579995 |
| Hif1a | 2.47E-43 | 0.875781379 | 0.487 | 0.217 | 4.52E-39 |
| Tut4 | 4.40E-67 | 0.86594285 | 0.759 | 0.477 | 8.05E-63 |
| Gimap6 | 5.74E-83 | 0.864273219 | 0.909 | 0.771 | 1.05E-78 |
| Cebpb | 3.46E-33 | 0.852258031 | 0.392 | 0.168 | 6.34E-29 |
| Nfkbia | 9.29E-33 | 0.848210278 | 0.734 | 0.554 | 1.70E-28 |
| Fam107b | 2.16E-66 | 0.839651989 | 0.71 | 0.381 | 3.95E-62 |
| Ccl3 | 1.52E-06 | 0.826360305 | 0.103 | 0.048 | 0.027858553 |
| P2ry10 | 6.82E-90 | 0.819430697 | 0.531 | 0.118 | 1.25E-85 |
| Ndrg3 | 2.89E-51 | 0.818911656 | 0.472 | 0.175 | 5.29E-47 |

avg_log2FC, log fold-change of the average expression between the two groups (positive values indicate that the gene is more highly expressed in CLP); pct.1, percent of CLP cells with detected expression of the gene; pct.2, percent of sham cells with detected expression the gene; p_val_adj, adjusted p-value based on Bonferroni correction using all genes in the dataset.

**Supplemental Table 5. Differentially-expressed genes of neutrophil subsets.**

| **cluster** | **gene** | **p_val** | **avg_log2FC** | **pct.1** | **pct.2** | **p_val_adj** |
| --- | --- | --- | --- | --- | --- | --- |
| 0 | Csf3 | 1.78E-31 | 0.728377853 | 0.711 | 0.576 | 1.94E-27 |
| 0 | Chka | 7.11E-35 | 0.665651236 | 0.699 | 0.53 | 7.75E-31 |
| 0 | Hmox1 | 1.67E-40 | 0.655825348 | 0.856 | 0.744 | 1.82E-36 |
| 0 | Hcar2 | 6.52E-61 | 0.574415779 | 0.976 | 0.903 | 7.11E-57 |
| 0 | Camp | 2.49E-15 | 0.533377901 | 0.39 | 0.281 | 2.71E-11 |
| 0 | Cstb | 6.56E-50 | 0.48667917 | 0.949 | 0.84 | 7.15E-46 |
| 0 | Gde1 | 6.99E-38 | 0.460673884 | 0.7 | 0.523 | 7.62E-34 |
| 0 | Gadd45g | 2.64E-07 | 0.429007636 | 0.144 | 0.091 | 0.002876204 |
| 0 | Cpeb4 | 3.18E-23 | 0.421189998 | 0.584 | 0.436 | 3.47E-19 |
| 0 | Syne1 | 1.09E-27 | 0.415085893 | 0.606 | 0.446 | 1.19E-23 |
| 0 | Suco | 5.37E-24 | 0.410342737 | 0.73 | 0.601 | 5.86E-20 |
| 0 | Dnajb9 | 5.46E-14 | 0.408795817 | 0.295 | 0.196 | 5.96E-10 |
| 0 | Il10 | 1.08E-08 | 0.406356497 | 0.643 | 0.586 | 0.000117326 |
| 0 | Ngp | 2.71E-08 | 0.405702369 | 0.852 | 0.814 | 0.000295549 |
| 0 | Rab7 | 5.24E-47 | 0.391501951 | 0.989 | 0.962 | 5.71E-43 |
| 0 | Ftl1 | 1.17E-17 | 0.391458052 | 0.999 | 1 | 1.27E-13 |
| 0 | Hilpda | 1.36E-17 | 0.38371904 | 0.744 | 0.649 | 1.48E-13 |
| 0 | Zfp292 | 4.84E-29 | 0.37838682 | 0.52 | 0.351 | 5.28E-25 |
| 0 | Sod2 | 3.05E-36 | 0.376626051 | 0.985 | 0.958 | 3.32E-32 |
| 0 | Slpi | 1.99E-27 | 0.376013447 | 0.996 | 0.987 | 2.17E-23 |
| 1 | Il1b | 4.74E-100 | 1.571680646 | 0.969 | 0.892 | 5.17E-96 |
| 1 | Pou2f2 | 1.73E-133 | 1.324874018 | 0.618 | 0.235 | 1.88E-129 |
| 1 | Emp3 | 1.54E-119 | 1.27552235 | 0.691 | 0.355 | 1.68E-115 |
| 1 | Ifitm1 | 3.58E-84 | 1.21034977 | 0.845 | 0.59 | 3.90E-80 |
| 1 | Gm19951 | 2.20E-155 | 1.153219957 | 0.401 | 0.062 | 2.40E-151 |
| 1 | Nr4a1 | 1.14E-129 | 1.070598319 | 0.77 | 0.421 | 1.24E-125 |
| 1 | Pglyrp1 | 8.36E-91 | 1.041200097 | 0.777 | 0.508 | 9.11E-87 |
| 1 | Prr13 | 4.94E-114 | 0.998351265 | 0.843 | 0.587 | 5.38E-110 |
| 1 | S100a6 | 1.24E-123 | 0.996185855 | 0.996 | 0.975 | 1.35E-119 |
| 1 | Klf2 | 9.00E-101 | 0.986545798 | 0.961 | 0.803 | 9.81E-97 |
| 1 | Gpx1 | 3.07E-96 | 0.962670289 | 0.505 | 0.188 | 3.35E-92 |
| 1 | Nfam1 | 1.19E-102 | 0.962494395 | 0.634 | 0.297 | 1.30E-98 |
| 1 | Zfp36l1 | 1.64E-74 | 0.951181682 | 0.696 | 0.446 | 1.78E-70 |
| 1 | H2-D1 | 1.46E-130 | 0.933144233 | 0.95 | 0.796 | 1.59E-126 |
| 1 | Adgre5 | 4.33E-148 | 0.913203947 | 0.973 | 0.783 | 4.72E-144 |
| 1 | Arf2 | 9.06E-114 | 0.90084876 | 0.791 | 0.455 | 9.87E-110 |
| 1 | Kras | 6.43E-116 | 0.778175973 | 0.814 | 0.506 | 7.01E-112 |
| 1 | Ly6g | 4.30E-55 | 0.774929415 | 0.42 | 0.186 | 4.69E-51 |
| 1 | Tnfaip2 | 7.72E-56 | 0.764944964 | 0.644 | 0.412 | 8.41E-52 |
| 1 | Flna | 8.60E-85 | 0.733044368 | 0.621 | 0.31 | 9.37E-81 |
| 2 | Saa3 | 1.73E-190 | 3.504449841 | 1 | 0.782 | 1.88E-186 |
| 2 | Orm1 | 6.06E-08 | 0.453428547 | 0.789 | 0.702 | 0.000660633 |
| 2 | Il10 | 1.81E-06 | 0.449728356 | 0.728 | 0.613 | 0.019783405 |
| 2 | Plin2 | 9.19E-12 | 0.366279844 | 0.974 | 0.921 | 1.00E-07 |
| 2 | Csf3 | 2.19E-07 | 0.338505418 | 0.76 | 0.653 | 0.002390027 |
| 2 | Sod2 | 4.99E-11 | 0.310237401 | 0.991 | 0.974 | 5.44E-07 |
| 2 | Lamp1 | 3.90E-11 | 0.306429886 | 0.874 | 0.734 | 4.25E-07 |
| 2 | Gde1 | 9.10E-11 | 0.297626016 | 0.775 | 0.622 | 9.91E-07 |
| 2 | Fnip2 | 5.16E-10 | 0.284290745 | 0.822 | 0.677 | 5.62E-06 |
| 2 | Cd63 | 5.00E-09 | 0.281475318 | 0.924 | 0.827 | 5.45E-05 |
| 2 | Rab7 | 1.36E-09 | 0.268323224 | 0.994 | 0.977 | 1.48E-05 |
| 2 | Txn1 | 1.92E-09 | 0.257532085 | 0.977 | 0.936 | 2.10E-05 |
| 2 | Ctsd | 1.82E-06 | 0.253948806 | 0.944 | 0.91 | 0.019828488 |
| 2 | Prdx5 | 4.00E-12 | 0.252016649 | 1 | 0.997 | 4.36E-08 |
| 3 | Edn1 | 2.93E-17 | 1.161315825 | 0.484 | 0.308 | 3.19E-13 |
| 3 | Rpsa | 1.13E-18 | 1.150496291 | 0.484 | 0.283 | 1.23E-14 |
| 3 | Rpl13 | 5.39E-17 | 1.102046963 | 0.59 | 0.403 | 5.88E-13 |
| 3 | Spp1 | 2.47E-10 | 1.070829568 | 0.292 | 0.162 | 2.69E-06 |
| 3 | Rpl32 | 2.29E-16 | 0.971122226 | 0.422 | 0.238 | 2.50E-12 |
| 3 | Rps24 | 1.57E-13 | 0.930077971 | 0.702 | 0.565 | 1.71E-09 |
| 3 | Rps20 | 2.55E-21 | 0.930024917 | 0.634 | 0.408 | 2.78E-17 |
| 3 | Rps19 | 9.75E-11 | 0.910932812 | 0.444 | 0.309 | 1.06E-06 |
| 3 | Rps8 | 5.68E-18 | 0.875709362 | 0.63 | 0.424 | 6.19E-14 |
| 3 | Rplp0 | 3.42E-19 | 0.874965157 | 0.714 | 0.538 | 3.73E-15 |
| 3 | Rps5 | 1.42E-15 | 0.836601742 | 0.457 | 0.273 | 1.55E-11 |
| 3 | Rps15a | 2.40E-21 | 0.822917421 | 0.596 | 0.378 | 2.61E-17 |
| 3 | Rps18 | 2.78E-19 | 0.818092499 | 0.317 | 0.14 | 3.03E-15 |
| 3 | Rpl12 | 5.41E-10 | 0.807767703 | 0.351 | 0.219 | 5.90E-06 |
| 3 | Rpl19 | 7.23E-16 | 0.799701635 | 0.674 | 0.509 | 7.88E-12 |
| 3 | Rpl8 | 5.92E-19 | 0.795104304 | 0.634 | 0.422 | 6.45E-15 |
| 3 | Rps4x | 1.51E-12 | 0.79146933 | 0.463 | 0.302 | 1.64E-08 |
| 3 | Rps3a1 | 2.21E-17 | 0.776995499 | 0.72 | 0.587 | 2.40E-13 |
| 3 | Rpl17 | 2.24E-23 | 0.770767263 | 0.807 | 0.636 | 2.44E-19 |
| 3 | Rps7 | 5.31E-16 | 0.756838794 | 0.627 | 0.426 | 5.79E-12 |

avg_log2FC, log fold-change of the average expression between subcluster X and other neutrophils (positive values indicate that the gene is more highly expressed in subcluster X); pct.1, percent of subcluster X cells with detected expression of the gene; pct.2, percent of all other neutrophils with detected expression of the gene; p_val_adj, adjusted p-value based on Bonferroni correction using all genes in the dataset.

**Supplemental Table 6. Differentially-expressed genes of macrophage subsets.**

| **cluster** | **gene** | **p_val** | **avg_log2FC** | **pct.1** | **pct.2** | **p_val_adj** |
| --- | --- | --- | --- | --- | --- | --- |
| 0 | Slpi | 0 | 2.492495003 | 1 | 0.936 | 0 |
| 0 | Chil3 | 2.98E-52 | 0.823970559 | 0.309 | 0.107 | 4.21E-48 |
| 0 | S100a4 | 2.10E-29 | 0.426426285 | 0.956 | 0.926 | 2.97E-25 |
| 0 | S100a6 | 1.17E-22 | 0.384446773 | 0.973 | 0.944 | 1.65E-18 |
| 0 | Fabp5 | 4.38E-11 | 0.353138003 | 0.913 | 0.877 | 6.19E-07 |
| 0 | Prtn3 | 2.15E-28 | 0.342573631 | 0.959 | 0.917 | 3.04E-24 |
| 0 | Apoc2 | 7.35E-07 | 0.335845335 | 0.81 | 0.806 | 0.010386628 |
| 0 | Cd52 | 1.97E-31 | 0.331517669 | 0.918 | 0.821 | 2.79E-27 |
| 0 | Tmem176b | 5.88E-18 | 0.305074304 | 0.824 | 0.764 | 8.31E-14 |
| 0 | Fabp4 | 4.43E-19 | 0.289232392 | 0.981 | 0.963 | 6.27E-15 |
| 0 | F7 | 1.86E-15 | 0.277215978 | 0.592 | 0.484 | 2.63E-11 |
| 0 | Lyz1 | 1.49E-10 | 0.276470212 | 0.935 | 0.935 | 2.11E-06 |
| 0 | Tmem176a | 1.63E-12 | 0.274570946 | 0.765 | 0.723 | 2.31E-08 |
| 0 | S100a11 | 1.27E-18 | 0.263805703 | 0.822 | 0.782 | 1.80E-14 |
| 0 | Saa3 | 9.17E-09 | 0.258630433 | 0.993 | 0.983 | 0.000129546 |
| 0 | Fabp7 | 1.13E-10 | 0.251365039 | 0.827 | 0.791 | 1.59E-06 |
| 0 | Ctsa | 4.12E-20 | -0.2501293 | 0.937 | 0.968 | 5.83E-16 |
| 0 | Top2a | 6.38E-18 | -0.256204001 | 0.051 | 0.14 | 9.02E-14 |
| 0 | Sirpa | 1.16E-12 | -0.257454855 | 0.531 | 0.649 | 1.63E-08 |
| 0 | Dek | 1.35E-10 | -0.25979828 | 0.799 | 0.838 | 1.91E-06 |
| 1 | Cxcl13 | 7.69E-53 | 1.462006297 | 0.533 | 0.322 | 1.09E-48 |
| 1 | Apoc1 | 5.87E-27 | 1.23668825 | 0.252 | 0.12 | 8.30E-23 |
| 1 | Vsig4 | 2.07E-28 | 1.016071952 | 0.458 | 0.319 | 2.93E-24 |
| 1 | Wnt2 | 2.57E-32 | 0.735824395 | 0.495 | 0.342 | 3.63E-28 |
| 1 | Tgfb2 | 1.43E-75 | 0.710015636 | 0.925 | 0.75 | 2.01E-71 |
| 1 | Timd4 | 6.83E-78 | 0.689259406 | 0.835 | 0.596 | 9.65E-74 |
| 1 | C4b | 5.21E-100 | 0.575346855 | 0.99 | 0.942 | 7.37E-96 |
| 1 | Cald1 | 3.61E-52 | 0.531115545 | 0.811 | 0.651 | 5.11E-48 |
| 1 | Scn1b | 8.47E-49 | 0.46222353 | 0.835 | 0.714 | 1.20E-44 |
| 1 | Cdkn2a | 2.46E-23 | 0.461963691 | 0.21 | 0.094 | 3.48E-19 |
| 1 | Asz1 | 1.79E-35 | 0.434934256 | 0.211 | 0.071 | 2.53E-31 |
| 1 | Cd63 | 2.87E-25 | 0.432151134 | 0.55 | 0.409 | 4.06E-21 |
| 1 | Marco | 9.48E-07 | 0.373893099 | 0.555 | 0.5 | 0.013403444 |
| 1 | Fscn1 | 3.38E-38 | 0.37194982 | 0.258 | 0.096 | 4.78E-34 |
| 1 | Timp2 | 2.78E-89 | 0.371775566 | 1 | 1 | 3.93E-85 |
| 1 | Trf | 1.19E-52 | 0.357667042 | 0.999 | 0.999 | 1.68E-48 |
| 1 | Wfdc17 | 3.66E-54 | 0.354953054 | 1 | 1 | 5.18E-50 |
| 1 | Ecm1 | 1.39E-57 | 0.354387655 | 0.999 | 0.997 | 1.96E-53 |
| 1 | Tln2 | 7.10E-33 | 0.339270692 | 0.39 | 0.21 | 1.00E-28 |
| 1 | Tcn2 | 1.09E-46 | 0.336974052 | 0.988 | 0.977 | 1.54E-42 |
| 2 | S100a9 | 2.40E-214 | 2.547914052 | 0.36 | 0.007 | 3.40E-210 |
| 2 | S100a8 | 6.83E-51 | 2.336623818 | 0.46 | 0.194 | 9.65E-47 |
| 2 | Cd74 | 9.65E-56 | 2.327536368 | 0.703 | 0.378 | 1.36E-51 |
| 2 | AW112010 | 3.30E-38 | 2.003703076 | 0.48 | 0.251 | 4.66E-34 |
| 2 | Hmox1 | 3.49E-42 | 2.003063787 | 0.775 | 0.551 | 4.94E-38 |
| 2 | Vcam1 | 2.57E-157 | 1.910936535 | 0.235 | 0.001 | 3.63E-153 |
| 2 | Hbb-bs | 4.89E-89 | 1.715136064 | 0.182 | 0.008 | 6.91E-85 |
| 2 | Marcks | 1.69E-40 | 1.570154444 | 0.475 | 0.235 | 2.39E-36 |
| 2 | Spic | 1.58E-123 | 1.450368786 | 0.192 | 0.002 | 2.23E-119 |
| 2 | Aif1 | 1.24E-44 | 1.345536842 | 0.329 | 0.104 | 1.76E-40 |
| 2 | H2-Aa | 2.92E-22 | 1.308304473 | 0.324 | 0.16 | 4.13E-18 |
| 2 | Fth1 | 2.44E-34 | 1.224825452 | 1 | 1 | 3.44E-30 |
| 2 | H2-Ab1 | 2.26E-14 | 1.21832582 | 0.331 | 0.204 | 3.19E-10 |
| 2 | Tmsb10 | 1.27E-47 | 1.2002328 | 0.93 | 0.773 | 1.80E-43 |
| 2 | H2-Eb1 | 3.65E-30 | 1.136158036 | 0.278 | 0.101 | 5.16E-26 |
| 2 | Cxcl2 | 4.23E-07 | 1.023639629 | 0.235 | 0.15 | 0.005976681 |
| 2 | Igkc | 9.40E-12 | 1.003348697 | 0.3 | 0.19 | 1.33E-07 |
| 2 | Mrc1 | 8.24E-81 | 1.00127552 | 0.283 | 0.039 | 1.17E-76 |
| 2 | Axl | 2.44E-46 | 1.000734881 | 0.249 | 0.057 | 3.45E-42 |
| 2 | Coro1a | 9.88E-18 | 0.997492755 | 0.405 | 0.257 | 1.40E-13 |
| 3 | Stmn1 | 6.52E-302 | 1.859156748 | 0.792 | 0.057 | 9.22E-298 |
| 3 | Mcm6 | 6.23E-221 | 1.762784121 | 0.947 | 0.185 | 8.80E-217 |
| 3 | Mcm3 | 4.84E-213 | 1.748578736 | 0.97 | 0.216 | 6.84E-209 |
| 3 | Lig1 | 3.40E-275 | 1.69354279 | 0.867 | 0.096 | 4.80E-271 |
| 3 | Mcm7 | 1.27E-299 | 1.655795061 | 0.909 | 0.097 | 1.79E-295 |
| 3 | Mcm5 | 1.58E-302 | 1.57783976 | 0.902 | 0.091 | 2.24E-298 |
| 3 | Hells | 0 | 1.55753183 | 0.867 | 0.07 | 0 |
| 3 | Dut | 3.26E-207 | 1.494369445 | 0.856 | 0.141 | 4.61E-203 |
| 3 | Nasp | 1.05E-155 | 1.4558384 | 0.92 | 0.27 | 1.49E-151 |
| 3 | Hmgb2 | 3.00E-98 | 1.4237625 | 0.977 | 0.68 | 4.25E-94 |
| 3 | Smc2 | 4.42E-205 | 1.399185866 | 0.867 | 0.146 | 6.24E-201 |
| 3 | Gmnn | 7.84E-172 | 1.371148952 | 0.852 | 0.18 | 1.11E-167 |
| 3 | Dnmt1 | 1.09E-147 | 1.30678711 | 0.867 | 0.224 | 1.53E-143 |
| 3 | Atad2 | 2.36E-171 | 1.287261844 | 0.83 | 0.161 | 3.33E-167 |
| 3 | Mcm4 | 1.49E-219 | 1.284181987 | 0.833 | 0.114 | 2.10E-215 |
| 3 | Pcna | 3.96E-112 | 1.276805559 | 0.894 | 0.353 | 5.59E-108 |
| 3 | Uhrf1 | 0 | 1.217110536 | 0.659 | 0.026 | 0 |
| 3 | Pclaf | 1.10E-203 | 1.214597832 | 0.394 | 0.011 | 1.56E-199 |
| 3 | Mcm2 | 1.42E-233 | 1.200304801 | 0.795 | 0.09 | 2.01E-229 |
| 3 | Top2a | 1.77E-229 | 1.197024608 | 0.674 | 0.057 | 2.50E-225 |

avg_log2FC, log fold-change of the average expression between subcluster X and other macrophages (positive values indicate that the gene is more highly expressed in subcluster X); pct.1, percent of subcluster X cells with detected expression of the gene; pct.2, percent of all other macrophages with detected expression of the gene; p_val_adj, adjusted p-value based on Bonferroni correction using all genes in the dataset.

**Supplemental Table 7.** **Differentially-expressed genes of B cell subsets.**

| **cluster** | **gene** | **p_val** | **avg_log2FC** | **pct.1** | **pct.2** | **p_val_adj** |
| --- | --- | --- | --- | --- | --- | --- |
| 0 | Ighd | 3.66E-136 | 1.227195216 | 0.825 | 0.281 | 4.67E-132 |
| 0 | Ebf1 | 1.94E-137 | 1.098451213 | 0.955 | 0.655 | 2.47E-133 |
| 0 | Mef2c | 6.79E-101 | 0.984726041 | 0.913 | 0.621 | 8.67E-97 |
| 0 | Fcer2a | 4.73E-100 | 0.931658193 | 0.625 | 0.135 | 6.05E-96 |
| 0 | Btg1 | 2.74E-99 | 0.894052428 | 0.907 | 0.593 | 3.50E-95 |
| 0 | BE692007 | 2.33E-63 | 0.799052494 | 0.7 | 0.353 | 2.97E-59 |
| 0 | H2-Aa | 1.15E-121 | 0.763950579 | 0.999 | 0.909 | 1.47E-117 |
| 0 | Vpreb3 | 7.85E-64 | 0.759676066 | 0.46 | 0.103 | 1.00E-59 |
| 0 | H2-Ab1 | 6.16E-109 | 0.756711358 | 0.999 | 0.906 | 7.87E-105 |
| 0 | Cd69 | 4.50E-41 | 0.750112814 | 0.507 | 0.237 | 5.75E-37 |
| 0 | Stk17b | 2.60E-64 | 0.732636903 | 0.742 | 0.403 | 3.32E-60 |
| 0 | H2-Ob | 5.42E-55 | 0.675603441 | 0.736 | 0.457 | 6.92E-51 |
| 0 | Shisa5 | 2.69E-63 | 0.665563889 | 0.903 | 0.715 | 3.43E-59 |
| 0 | Ltb | 5.76E-63 | 0.655958971 | 0.939 | 0.707 | 7.35E-59 |
| 0 | Cr2 | 9.90E-41 | 0.654901488 | 0.471 | 0.175 | 1.26E-36 |
| 0 | Sesn1 | 7.57E-45 | 0.576233719 | 0.572 | 0.263 | 9.67E-41 |
| 0 | Ralgps2 | 4.25E-37 | 0.568263716 | 0.776 | 0.576 | 5.43E-33 |
| 0 | Cd55 | 3.37E-34 | 0.554549562 | 0.578 | 0.329 | 4.30E-30 |
| 0 | Rhoh | 8.80E-42 | 0.5493047 | 0.619 | 0.338 | 1.12E-37 |
| 0 | Fchsd2 | 5.00E-35 | 0.545959982 | 0.546 | 0.291 | 6.38E-31 |
| 1 | S100a6 | 1.65E-213 | 3.797542524 | 0.785 | 0.139 | 2.11E-209 |
| 1 | Crip1 | 1.44E-114 | 2.227659214 | 0.911 | 0.703 | 1.84E-110 |
| 1 | Vim | 4.55E-132 | 1.869891575 | 0.807 | 0.353 | 5.81E-128 |
| 1 | Ahnak | 1.38E-178 | 1.726725853 | 0.716 | 0.108 | 1.76E-174 |
| 1 | Plac8 | 3.50E-159 | 1.614142403 | 0.945 | 0.524 | 4.46E-155 |
| 1 | S100a4 | 5.00E-141 | 1.477882398 | 0.49 | 0.032 | 6.38E-137 |
| 1 | Iglc1 | 9.35E-47 | 1.372145834 | 0.822 | 0.504 | 1.19E-42 |
| 1 | Lgals1 | 9.12E-117 | 1.242555886 | 0.594 | 0.124 | 1.16E-112 |
| 1 | Tagln2 | 6.24E-98 | 1.189117823 | 0.748 | 0.316 | 7.97E-94 |
| 1 | Lyz2 | 5.51E-73 | 1.158954188 | 0.72 | 0.318 | 7.04E-69 |
| 1 | Rassf4 | 1.04E-155 | 1.041391593 | 0.634 | 0.086 | 1.33E-151 |
| 1 | Lsp1 | 5.33E-86 | 0.992002515 | 0.894 | 0.659 | 6.80E-82 |
| 1 | Zcwpw1 | 3.12E-73 | 0.943667056 | 0.329 | 0.04 | 3.99E-69 |
| 1 | Itgb1 | 3.57E-154 | 0.942421624 | 0.584 | 0.058 | 4.56E-150 |
| 1 | Gm15987 | 1.20E-85 | 0.918920394 | 0.673 | 0.259 | 1.53E-81 |
| 1 | Ccnd2 | 2.64E-91 | 0.871841398 | 0.557 | 0.127 | 3.37E-87 |
| 1 | Zbtb20 | 9.30E-56 | 0.843975867 | 0.622 | 0.282 | 1.19E-51 |
| 1 | Dnajc7 | 8.84E-69 | 0.839252913 | 0.737 | 0.418 | 1.13E-64 |
| 1 | Apoe | 9.38E-55 | 0.832190145 | 0.658 | 0.279 | 1.20E-50 |
| 1 | Cd2 | 3.09E-78 | 0.831415942 | 0.745 | 0.356 | 3.95E-74 |
| 2 | Hmgb2 | 3.77E-11 | 1.796773646 | 0.553 | 0.335 | 4.82E-07 |
| 2 | Ncl | 7.40E-48 | 1.795346832 | 0.992 | 0.753 | 9.45E-44 |
| 2 | Mif | 4.37E-48 | 1.523854646 | 0.911 | 0.446 | 5.58E-44 |
| 2 | Npm1 | 1.23E-50 | 1.497430814 | 1 | 0.877 | 1.58E-46 |
| 2 | Hist1h1b | 3.13E-13 | 1.367091496 | 0.309 | 0.106 | 4.00E-09 |
| 2 | Eif5a | 9.42E-43 | 1.34247124 | 0.935 | 0.642 | 1.20E-38 |
| 2 | Ran | 8.08E-53 | 1.33208965 | 0.959 | 0.495 | 1.03E-48 |
| 2 | Nme1 | 8.39E-41 | 1.318292965 | 0.902 | 0.521 | 1.07E-36 |
| 2 | Stmn1 | 5.52E-33 | 1.317284849 | 0.366 | 0.069 | 7.05E-29 |
| 2 | Pclaf | 4.71E-53 | 1.313184313 | 0.276 | 0.019 | 6.01E-49 |
| 2 | Ranbp1 | 2.66E-47 | 1.302879293 | 0.878 | 0.386 | 3.40E-43 |
| 2 | Ptma | 3.69E-53 | 1.229332857 | 1 | 0.974 | 4.71E-49 |
| 2 | Anp32b | 2.74E-50 | 1.2179157 | 0.967 | 0.581 | 3.49E-46 |
| 2 | Hsp90ab1 | 1.21E-44 | 1.205250789 | 1 | 0.98 | 1.55E-40 |
| 2 | H2afz | 9.13E-15 | 1.165213967 | 0.813 | 0.616 | 1.17E-10 |
| 2 | Mki67 | 9.26E-22 | 1.158769162 | 0.228 | 0.039 | 1.18E-17 |
| 2 | Ybx1 | 8.15E-36 | 1.130917263 | 0.984 | 0.788 | 1.04E-31 |
| 2 | Tubb5 | 1.16E-22 | 1.128842101 | 0.846 | 0.536 | 1.48E-18 |
| 2 | Hspd1 | 4.44E-38 | 1.127743569 | 0.837 | 0.384 | 5.67E-34 |
| 2 | Top2a | 9.32E-32 | 1.097150007 | 0.252 | 0.031 | 1.19E-27 |
| 3 | Ighg2c | 1.25E-21 | 6.916069568 | 0.123 | 0.005 | 1.60E-17 |
| 3 | Jchain | 5.50E-184 | 6.134603795 | 0.982 | 0.04 | 7.02E-180 |
| 3 | Ighg2b | 8.37E-14 | 5.453388798 | 0.123 | 0.01 | 1.07E-09 |
| 3 | Igkc | 5.02E-30 | 4.907936235 | 0.947 | 0.976 | 6.41E-26 |
| 3 | Ighm | 6.75E-17 | 4.662495598 | 0.842 | 0.994 | 8.61E-13 |
| 3 | Slpi | 1.80E-14 | 4.476545564 | 0.526 | 0.196 | 2.29E-10 |
| 3 | Iglv1 | 7.61E-114 | 4.452846746 | 0.93 | 0.075 | 9.71E-110 |
| 3 | Hsp90b1 | 1.76E-40 | 3.586341473 | 1 | 0.525 | 2.25E-36 |
| 3 | Xbp1 | 1.45E-84 | 3.391364173 | 1 | 0.149 | 1.85E-80 |
| 3 | Iglc1 | 2.58E-13 | 3.370963484 | 0.86 | 0.592 | 3.29E-09 |
| 3 | Mzb1 | 5.94E-31 | 3.246049615 | 0.982 | 0.76 | 7.58E-27 |
| 3 | Txndc5 | 2.49E-57 | 3.19999911 | 0.965 | 0.232 | 3.18E-53 |
| 3 | Ly6c2 | 1.89E-100 | 3.035167127 | 0.772 | 0.052 | 2.41E-96 |
| 3 | Sec11c | 4.83E-38 | 2.996675133 | 0.982 | 0.564 | 6.17E-34 |
| 3 | Ssr4 | 2.14E-40 | 2.994682609 | 0.982 | 0.548 | 2.73E-36 |
| 3 | Mt1 | 1.12E-42 | 2.970831059 | 0.404 | 0.034 | 1.43E-38 |
| 3 | Manf | 1.05E-46 | 2.810250039 | 0.982 | 0.335 | 1.34E-42 |
| 3 | Rexo2 | 3.20E-49 | 2.678685578 | 0.947 | 0.262 | 4.08E-45 |
| 3 | Edem1 | 1.28E-99 | 2.611978705 | 0.965 | 0.101 | 1.63E-95 |
| 3 | Fkbp2 | 3.37E-69 | 2.496660353 | 0.965 | 0.177 | 4.30E-65 |

avg_log2FC, log fold-change of the average expression between subcluster X and other B cells (positive values indicate that the gene is more highly expressed in subcluster X); pct.1, percent of subcluster X cells with detected expression of the gene; pct.2, percent of all other B cells with detected expression of the gene; p_val_adj, adjusted p-value based on Bonferroni correction using all genes in the dataset.

**Supplemental Table 8.** **Differentially-expressed genes of T cell subsets.**

| **cluster** | **gene** | **p_val** | **avg_log2FC** | **pct.1** | **pct.2** | **p_val_adj** |
| --- | --- | --- | --- | --- | --- | --- |
| 0 | Emb | 3.92E-101 | 1.261642778 | 0.922 | 0.708 | 4.94E-97 |
| 0 | Vps37b | 5.40E-94 | 0.98579564 | 0.833 | 0.364 | 6.81E-90 |
| 0 | Ifngr1 | 3.94E-77 | 0.979228852 | 0.875 | 0.49 | 4.97E-73 |
| 0 | Junb | 6.08E-60 | 0.882353573 | 0.809 | 0.447 | 7.67E-56 |
| 0 | Fam241a | 3.48E-40 | 0.864080248 | 0.519 | 0.243 | 4.39E-36 |
| 0 | Ptpn22 | 9.26E-63 | 0.852061104 | 0.827 | 0.498 | 1.17E-58 |
| 0 | Gramd3 | 2.42E-59 | 0.781711935 | 0.767 | 0.41 | 3.05E-55 |
| 0 | Klhl6 | 3.64E-46 | 0.766321688 | 0.684 | 0.367 | 4.60E-42 |
| 0 | 4932438A13Rik | 1.63E-44 | 0.680780682 | 0.746 | 0.434 | 2.05E-40 |
| 0 | Zeb1 | 2.45E-49 | 0.679181959 | 0.65 | 0.304 | 3.10E-45 |
| 0 | Gimap5 | 4.88E-47 | 0.677107015 | 0.769 | 0.445 | 6.16E-43 |
| 0 | Il7r | 4.94E-39 | 0.67041589 | 0.907 | 0.767 | 6.23E-35 |
| 0 | Cblb | 4.48E-55 | 0.63774918 | 0.664 | 0.289 | 5.65E-51 |
| 0 | Tut4 | 1.45E-44 | 0.631809879 | 0.791 | 0.51 | 1.83E-40 |
| 0 | Ramp3 | 9.74E-55 | 0.624460558 | 0.38 | 0.083 | 1.23E-50 |
| 0 | Gimap6 | 9.15E-50 | 0.620546845 | 0.938 | 0.785 | 1.15E-45 |
| 0 | Bcl2l11 | 6.84E-54 | 0.618618984 | 0.573 | 0.215 | 8.63E-50 |
| 0 | Satb1 | 1.41E-30 | 0.615747133 | 0.877 | 0.751 | 1.78E-26 |
| 0 | Txnip | 5.23E-23 | 0.603496968 | 0.845 | 0.771 | 6.60E-19 |
| 0 | Socs3 | 2.19E-43 | 0.599056868 | 0.567 | 0.24 | 2.76E-39 |
| 1 | Igfbp4 | 4.92E-55 | 1.044086808 | 0.597 | 0.243 | 6.20E-51 |
| 1 | Tspan32 | 4.01E-38 | 0.645486027 | 0.624 | 0.326 | 5.06E-34 |
| 1 | Actb | 1.41E-56 | 0.638938561 | 1 | 0.997 | 1.77E-52 |
| 1 | Uba52 | 3.53E-66 | 0.610956018 | 1 | 0.995 | 4.45E-62 |
| 1 | Cd52 | 4.54E-43 | 0.60682576 | 1 | 0.881 | 5.72E-39 |
| 1 | Ly6c1 | 2.13E-47 | 0.567082171 | 0.281 | 0.045 | 2.68E-43 |
| 1 | Tmsb4x | 2.61E-45 | 0.54699222 | 1 | 0.999 | 3.29E-41 |
| 1 | Ifi27l2a | 1.20E-25 | 0.527262421 | 0.816 | 0.595 | 1.51E-21 |
| 1 | Trbc2 | 1.31E-28 | 0.526117593 | 0.979 | 0.916 | 1.65E-24 |
| 1 | Limd2 | 1.58E-31 | 0.518914855 | 0.94 | 0.799 | 1.99E-27 |
| 1 | Chd3 | 2.07E-28 | 0.51216116 | 0.666 | 0.407 | 2.61E-24 |
| 1 | Emp3 | 4.16E-26 | 0.500585898 | 0.779 | 0.546 | 5.24E-22 |
| 1 | Pfn1 | 2.61E-39 | 0.494533758 | 0.991 | 0.913 | 3.29E-35 |
| 1 | Dnaja1 | 8.48E-24 | 0.466378395 | 0.892 | 0.777 | 1.07E-19 |
| 1 | Ms4a6b | 1.78E-23 | 0.465001383 | 0.912 | 0.769 | 2.24E-19 |
| 1 | Tesc | 2.61E-38 | 0.464817576 | 0.426 | 0.147 | 3.29E-34 |
| 1 | Tmsb10 | 3.39E-46 | 0.4646259 | 1 | 0.997 | 4.28E-42 |
| 1 | Cd3g | 6.07E-32 | 0.459307131 | 0.968 | 0.781 | 7.66E-28 |
| 1 | Bcl11b | 2.25E-18 | 0.457749302 | 0.744 | 0.573 | 2.84E-14 |
| 1 | Cd4 | 3.34E-35 | 0.457166024 | 0.479 | 0.188 | 4.22E-31 |
| 2 | Ccl5 | 6.00E-112 | 3.15627594 | 0.669 | 0.141 | 7.57E-108 |
| 2 | S100a6 | 6.15E-109 | 1.951332057 | 0.644 | 0.128 | 7.76E-105 |
| 2 | Ly6c2 | 2.03E-86 | 1.900748246 | 0.596 | 0.154 | 2.56E-82 |
| 2 | S100a4 | 1.45E-79 | 1.737863568 | 0.393 | 0.048 | 1.83E-75 |
| 2 | Lgals1 | 1.15E-84 | 1.720068684 | 0.698 | 0.227 | 1.45E-80 |
| 2 | Nkg7 | 3.35E-83 | 1.574984477 | 0.76 | 0.328 | 4.22E-79 |
| 2 | AW112010 | 1.43E-91 | 1.471634167 | 0.927 | 0.598 | 1.81E-87 |
| 2 | S100a10 | 6.44E-88 | 1.437697825 | 0.929 | 0.549 | 8.12E-84 |
| 2 | Crip1 | 8.08E-56 | 1.228799053 | 0.932 | 0.651 | 1.02E-51 |
| 2 | Ahnak | 7.29E-89 | 1.188762619 | 0.59 | 0.132 | 9.20E-85 |
| 2 | Itgb1 | 1.80E-79 | 1.082128462 | 0.551 | 0.121 | 2.26E-75 |
| 2 | Id2 | 1.43E-32 | 1.082070342 | 0.537 | 0.261 | 1.81E-28 |
| 2 | Tmsb4x | 5.90E-99 | 1.068673864 | 1 | 0.999 | 7.44E-95 |
| 2 | Klrk1 | 2.50E-80 | 1.023892596 | 0.339 | 0.028 | 3.15E-76 |
| 2 | Vim | 6.17E-34 | 1.010066524 | 0.681 | 0.404 | 7.78E-30 |
| 2 | S100a11 | 3.09E-59 | 0.997167169 | 0.734 | 0.337 | 3.89E-55 |
| 2 | Il2rb | 2.37E-68 | 0.967505987 | 0.757 | 0.294 | 2.99E-64 |
| 2 | Hmgb2 | 2.26E-17 | 0.92208014 | 0.701 | 0.529 | 2.85E-13 |
| 2 | Trdc | 3.52E-17 | 0.889412886 | 0.133 | 0.029 | 4.44E-13 |
| 2 | Ctsw | 2.63E-67 | 0.854322545 | 0.593 | 0.178 | 3.31E-63 |
| 3 | Cd8b1 | 5.98E-171 | 2.025017805 | 0.972 | 0.253 | 7.54E-167 |
| 3 | Cd8a | 4.41E-156 | 1.376910375 | 0.861 | 0.149 | 5.56E-152 |
| 3 | Dapl1 | 5.21E-49 | 1.196627892 | 0.611 | 0.224 | 6.57E-45 |
| 3 | Ccr9 | 6.58E-101 | 0.663319374 | 0.444 | 0.036 | 8.30E-97 |
| 3 | Arl4c | 1.28E-39 | 0.658537869 | 0.885 | 0.545 | 1.61E-35 |
| 3 | Nkg7 | 3.21E-52 | 0.629123065 | 0.851 | 0.33 | 4.05E-48 |
| 3 | Uba52 | 9.99E-51 | 0.604357645 | 1 | 0.995 | 1.26E-46 |
| 3 | Tubb5 | 1.94E-35 | 0.599188706 | 0.844 | 0.539 | 2.45E-31 |
| 3 | Cd52 | 2.21E-31 | 0.587493397 | 0.99 | 0.894 | 2.79E-27 |
| 3 | Dnajc15 | 1.11E-34 | 0.581093727 | 0.781 | 0.437 | 1.39E-30 |
| 3 | Epsti1 | 9.82E-27 | 0.578101846 | 0.733 | 0.434 | 1.24E-22 |
| 3 | Thy1 | 3.38E-34 | 0.556899346 | 0.823 | 0.441 | 4.26E-30 |
| 3 | BE692007 | 7.03E-26 | 0.553069959 | 0.705 | 0.381 | 8.86E-22 |
| 3 | Cd3g | 1.51E-28 | 0.493023088 | 0.979 | 0.796 | 1.90E-24 |
| 3 | Rgcc | 5.71E-35 | 0.490530433 | 0.455 | 0.147 | 7.20E-31 |
| 3 | Dynll1 | 6.34E-25 | 0.488412565 | 0.809 | 0.483 | 8.00E-21 |
| 3 | Klf2 | 1.01E-19 | 0.483812296 | 0.951 | 0.761 | 1.28E-15 |
| 3 | Sell | 1.03E-18 | 0.470964187 | 0.74 | 0.481 | 1.30E-14 |
| 3 | Klk8 | 4.46E-17 | 0.460653764 | 0.688 | 0.468 | 5.63E-13 |
| 3 | Tmsb10 | 2.96E-34 | 0.455646461 | 1 | 0.997 | 3.73E-30 |

avg_log2FC, log fold-change of the average expression between subcluster X and other T cells (positive values indicate that the gene is more highly expressed in subcluster X); pct.1, percent of subcluster X cells with detected expression of the gene; pct.2, percent of all other T cells with detected expression of the gene; p_val_adj, adjusted p-value based on Bonferroni correction using all genes in the dataset.
